# CREPT promotes LUAD progression by enhancing the CDK9 and RNAPII assembly to promote ERK-driven gene transcription

**DOI:** 10.7150/thno.115572

**Published:** 2025-07-25

**Authors:** Mengdi Li, Yuting Lin, Jiayu Wang, He Yang, Danhui Ma, Ye Tian, Yi Wang, Liu Yang, Umar Farooq, Yinyin Wang, Fangli Ren, Jian Sheng, Guoqing Zhang, Liang Chen, Jun Li, Xiangnan Li, Zhijie Chang

**Affiliations:** 1 State Key Laboratory of Membrane Biology, School of Basic Medical Sciences, School of Medicine, Tsinghua University, Beijing, 100084, China.; 2 Beijing Tsinghua Changgung Hospital, School of Clinical Medicine, Tsinghua University, Beijing, 102218, China.; 3 Institute for Organ Transplant and Bionic Medicine, Tsinghua University, Beijing, 102218, China; 4 Key Laboratory of Industrial Biocatalysis, Ministry of Education of China, Department of Chemical Engineering, Tsinghua University, Beijing, 100084, China.; 5 Jinfeng Laboratory, Chongqing, 401329, China.; 6 Department of science and education department, The Second Affiliated Hospital of Jiaxing University, Jiaxing, 314000, China; 7 Thoracic Surgery Department, First Affiliated Hospital of Zhengzhou University, Zhengzhou, 450052, China.; 8 Institute of Life and Health Engineering, College of Life Science and Technology, Jinan University, Guangzhou, 510632, China.; 9 Heya Pharmaceutical Technology Company, Beijing, 100176, China.

**Keywords:** Lung adenocarcinoma (LUAD), Kirsten rat sarcoma proto-oncogene (KRAS), Cell cycle-related and expression elevated protein in tumor (CREPT), Extracellular signal-regulated kinase (ERK), Cyclin-dependent kinase 9 (CDK9)

## Abstract

**Background:** Despite advancements in EGFR- and KRAS-targeted therapies for lung adenocarcinoma (LUAD), novel targets are needed for patients unresponsive or resistant to current treatments. This study demonstrates the critical role of CREPT in modulating ERK-downstream gene transcription in LUAD progression.

**Methods:** CREPT expression and function were investigated using human LUAD tissues, EGFR/KRAS mutant LUAD cell lines, and mouse models. Micro-CT was used to monitor tumor progression. Adeno-associated virus (AAV)-mediated CREPT depletion was employed as a therapeutic strategy. RNA sequencing and luciferase reporter assays identified differentially expressed genes (DEGs) and affected signaling pathways. Protein interactions and CDK9 occupancy were assessed using multiplex immunofluorescence, immunoprecipitation, and chromatin immunoprecipitation (ChIP).

**Results:** CREPT overexpression correlated with poor LUAD patient survival and enhanced tumorigenesis in EGFR or KRAS mutant LUAD cells. *CREPT* deletion impaired LUAD initiation and progression in the CC10-rtTA;TetO-*KRAS^G12D^* mouse model. Mechanistically, CREPT promoted CDK9 assembly with RNA polymerase II (RNAPII) following ERK activation, enhancing transcription of malignancy-related genes downstream of KRAS-ERK-Elk-1 signaling. CREPT depletion and the mutants R106A and S134A disrupting CREPT-RNAPII interaction reduced CDK9 occupancy at Elk-1 downstream gene promoters and their expression. Targeting CREPT in both CC10-rtTA;TetO-*KRAS^G12D^* and xenograft mouse models resulted in tumor growth arrest. Furthermore, in a humanized mouse model, AAV-mediated CREPT silencing inhibited tumor progression and showed synergistic potential with pembrolizumab.

**Conclusion:** Our findings highlight CREPT as a pivotal regulator of LUAD progression and suggest it could be a potential therapeutic target for patients with EGFR or KRAS mutations insensitive or resistant to targeted therapies.

## Background

Lung cancer remains the most prevalent malignancy worldwide and is the leading cause of cancer-related mortality 1. Non-small cell lung cancer (NSCLC) accounts for the majority of lung cancer cases, with approximately 50% being diagnosed as lung adenocarcinoma (LUAD) and about 30% as lung squamous cell carcinoma (LUSC). 2. The prognosis for patients with metastatic NSCLC remains poor, with a five-year overall survival (OS) rate of only 6% 3. Recent advances in molecular biology have identified key genetic drivers in NSCLC include alterations in epidermal growth factor receptor (EGFR) mutations and Kirsten rat sarcoma proto-oncogene (KRAS), present in approximately 17% and 25-30% of the cases, respectively. Despite this advancement and available drugs developed to target EGFR or KRAS mutations, clinical outcomes have remained unsatisfactory 4.

The roles of EGFR and KRAS mutations in promoting tumorigenesis have been extensively investigated. The majority of EGFR mutations in lung cancer are found in the tyrosine kinase domain involving exon 19 deletions (E19del, about 45%) and the L858R point mutation in exon 21 (about 40%) 5. These mutations lead to constitutive activation of the EGFR, triggering excessive signaling through multiple downstream pathways, including the mitogen-activated protein kinase (MAPK)-extracellular signal-regulated kinase (ERK), and phosphoinositide 3-kinase (PI3K) signaling pathways 6. Simultaneously, KRAS mutations, predominantly occurring at codons 12, 13, or 61, accelerate the GDP-GTP exchange cycle, leading to aberrant signaling through pathways such as MAPK-ERK, PI3K, and Ras-like (Ral) 7. However, a recent study on acute KRAS suppression in KRAS-mutant cells indicates that aberrant KRAS signaling drives cancer growth primarily through the MAPK-ERK pathway, influencing cell cycle progression at multiple levels including gene transcription and protein phosphorylation 8. Nevertheless, both EGFR and KRAS mutations have been attributed to the activation of the MAPK-ERK signaling, which further promote the downstream gene expression to exacerbate the progression of the disease. In this context, current therapeutic strategies for LUAD primarily target EGFR or KRAS mutations, as well as components of their downstream pathways. Unfortunately, the current inhibitors have shown limited efficacy in the clinical treatment due to inherent or acquired drug resistance 9. There is an urgent need for novel therapeutic targets, particularly for patients who are either insensitive or have developed resistance to existing targeted therapies.

Cyclin-dependent kinase 9 (CDK9) has emerged as a crucial transcriptional regulator implicated in various oncogenic pathways, contributing to tumorigenesis and tumor progression 10, 11. As the catalytic subunit of the positive transcription elongation factor (P-TEFb), CDK9 facilitates the release of promoter-proximal paused RNA polymerase II (RNAPII) and accelerates productive transcriptional elongation 12. The MAPK-ERK pathway is essential for the regulation of CDK9-mediated transcription. ERK1/2 directly interacts with CDK9 in the nucleus *via* specific ERK docking motifs, primarily the D domain 13. This interaction facilitates CDK9 recruitment to the MYC promoter, enhancing RNAPII progression and consequently promoting MYC transcription 13. *In vitro* studies have shown that CDK9 inhibitors suppress tumor cell growth and stemness while inducing apoptosis in NSCLC 14, 15. Notably, CDK9 inhibitors showed promising efficacy against KRAS-mutant and EGFR-mutant NSCLCs, including those that have developed resistance to targeted therapies such as EGFR and KRAS^G12C^ inhibitors 15, 16, 17. The initial clinical trial of a CDK9 inhibitor, VIP152, demonstrated potential efficacy in patients with advanced malignancies including lymphoma and solid tumors 18. Furthermore, CDK9 inhibition was found to sensitize tumors to immune checkpoint blockade (ICB) therapy, with a synergistic effect in cancer treatment 12, 19. However, it is noteworthy that CDK9 mutations or amplifications rarely occur in cancer patients, especially in NSCLC 11, raising the concerns about the specificity of CDK9 inhibitors and their potential impact on normal cells. Consequently, achieving a therapeutic dose that balances efficacy with acceptable side effects has proven challenging. Furthermore, the identification of reliable biomarkers to predict which patients would benefit most from CDK9 inhibition remains elusive.

Previous studies demonstrated that CREPT (cell cycle-related and expression elevated protein in tumor, also named RPRD1B) was highly expressed in various tumors including NSCLC 20, 21, 22. Accumulating evidence has revealed the multifaceted roles of CREPT in tumorigenesis through transcriptional regulation, DNA damage response, and R-loop formation. At the transcriptional level, CREPT participates in the regulation of Wnt signaling pathway, which is crucial for colorectal cancer development 23. It directly interacts with the C-terminal domain (CTD) of RNAPII and recruits other factors, such as phosphatase RNA polymerase II-associated protein 2 and histone deacetylase 1, to modulate CTD modifications and gene transcription 24, 25. Furthermore, CREPT influences transcription termination by interacting with Xrn2, a key 5′-3′ exoribonuclease in the "torpedo model" of transcription termination 26, 27. Beyond its transcriptional functions, CREPT plays a pivotal role in maintaining genomic stability through diverse mechanisms. It enhances non-homologous end joining (NHEJ) repair of DNA double-strand breaks (DSBs) by interacting with key proteins such as Ku70, Ku86, and Artemis 26. CREPT depletion leads to R-loop accumulation, potentially causing deleterious DSBs due to replication fork collisions. Additionally, CREPT mediates homologous recombination repair by regulating *CDK1* expression in breast cancers 28. Furthermore, CREPT stabilizes core mismatch repair proteins, thus preserving microsatellite stability 29. These diverse functions of CREPT in tumor cells underscore its potential as a therapeutic target in cancer research.

Overexpression of CREPT was shown to promote tumorigenesis by modulating the transcription of cell cycle-related genes in NSCLC cells 21, 22. However, the precise role and underlying molecular mechanisms of CREPT in NSCLC development remain to be fully elucidated. Our study addresses this knowledge gap by revealing that CREPT was specifically upregulated in LUAD, and promoted the association of CDK9 with RNAPII to regulate gene transcription during the disease progression. We have uncovered a novel molecular mechanism in which CREPT and CDK9 collaboratively promote the progression of LUAD. Importantly, targeting CREPT showed significant anti-tumor effects in both the CC10-rtTA;TetO*-KRAS^G12D^* and xenograft mouse models. These findings collectively underscore the robust anti-tumor activity of CREPT inhibition and suggest its potential as a novel therapeutic strategy.

## Methods

### Cell culture

HEK-293T (RRID:CVCL_0063) and A549 (RRID:CVCL_0023) cell lines were cultured in Dulbecco's Modified Eagle Medium (DMEM) medium containing 10% fetal bovine serum (ExCell Bio). The H441 (RRID:CVCL_1561), PC-9 (RRID:CVCL_B260), PC-9/GR (resistant to gefitinib) and H1975 (RRID:CVCL_1511) cell lines were grown in Roswell Park Memorial Institute (RPMI) 1640 medium. SK-LU-1 (RRID:CVCL_0629) cells were cultured in Minimum Essential Medium (MEM) medium (Macgene Biotechnology). All cell culture media were supplemented with 10% fetal bovine serum (Gibco, USA), 100 U/mL of penicillin, and 100 mg/mL of streptomycin. Cells were maintained in a humidified incubator with 5% CO_2_ at 37 °C. All cell lines were authenticated by short tandem repeat (STR) profiling at the beginning of the study and every six months thereafter. Cells were regularly tested for mycoplasma contamination using a PCR-based detection kit every three months. Only mycoplasma-free cells with confirmed identities were used in experiments.

### Cell line establishment and transfection of siRNAs in cells

CREPT depletion was achieved using small hairpin RNA (sh-RNA) or small interfering RNA (siRNA) targeting CREPT. The shCREPT-1 and shCREPT-2 sequences are shown below: shCREPT-1: 5'-CCGGCGGCAGCAGTATATTCTGAAACTCGA GTTTCAGAATATACTGCTGCCGTTTTTTG-3'; shCREPT-2: 5'-CCGGGCACG AAGATTAGGTGCATTTCTCGAGAAATGCACCTAATCTTCGTGCTTTTTTG-3'. The si-RNA sequences targeting CREPT are shown below: siCtrl: 5'-UUCUCCGAAGUCACGUTT-3' and 5'-ACGUGACUUCGGAGAATT-3'; siCREPT-1: 5'-GUCUGUUACUAGCAGAAUATT-3' and 5'-UAUUCUGCUAGUAACAGACTT-3'; siCREPT-2: 5'-GACCUGAAUUCACUAGAGATT-3' and 5'-UCUCUAGUGAAUUCAGGUCTT-3'; siCREPT-3: 5'-GGUCCGCAAGGAACUGAAATT-3' and 5'-UUUCAGUUCCUUGCGGACCTT-3'. CREPT overexpression (CREPT-OE) cell lines were obtained by lentivirus infection. The lentivirus packed pcDH-EGFP-HA-CREPT and selected by EGFP.

KRAS depletion was achieved using the siRNAs showing below: siKRAS-1: 5'-CCUUGACGAUACAGCUAAUTT-3' and 5'-AUUAGCUGUAUCGUCAAGGTT-3'; siKRAS-2: 5'-UCUCGACACAGCAGGUCAATT-3' and 5'-UUGACCUGCUGUGUCGAGATT-3'; siKRAS-3: 5'-GGACUUAGCAAGAAGUUAUTT-3' and 5'-AUAACUUCUUGCUAAGUCCTT-3'. CDK9 depletion was achieved using the siRNAs showing below: siCDK9-1: 5'-GGGAGAUCAAGAUCCUUCATT-3' and 5'-UGAAGGAUCUUGAUCUCCCTT-3'; siCDK9-2: 5'-GCUGCUAAUGUGCUUAUCATT-3' and 5'-UGAUAAGCACAUUAGCAGCTT-3'; siCDK9-3: 5'-GGCCAAACGUGGACAACUATT-3' and 5'-UAGUUGUCCACGUUUGGCCTT-3'.

### Mouse model

*CREPT^flox/flox^* and Tg-*CREPT* mice were constructed previously by our laboratory 30. Tg-*CREPT* mice were crossed with EIIα-Cre mice for CREPT overexpression. The TetO-*KRAS^G12D^* mice are CC10-rtTA;TetO-*KRAS^G12D^* 31, kindly provided by Prof. Chen Liang. The background of *CREPT^flox/flox^* and Tg-*CREPT* mice was C57BL/6, and the background of TetO-*KRAS^G12D^* mice was 129/Sv. We utilized three genetically engineered mouse models, including TetO-*KRAS^G12D^*;*CREPT^flox/flox^*, TetO-*KRAS^G12D^*;Tg-*CREPT* and CreERT2+/-;TetO-*KRAS^G12D^*;*CREPT^flox/flox^* mice that allows for conditional deletion of *CREPT* or *CREPT* overexpression in TetO-*KRAS^G12D^* mice.

All mice used in this experiment were raised on the animal platform of Tsinghua University. The experimental protocol was approved by the IACUC (Institutional Animal Care and Use Committee) of Tsinghua University. The ethical review approval number was 20-CZJ and 24-CJZ4. The mice were maintained in a constant temperature environment of 22-26 °C, with a light/dark cycle of 12/12 h, with no more than 6 mice per cage. All mice were free to access feed and water. Mice of both sexes were included in the study, with an age range of 6-10 weeks at the start of experiments. Mice showing any signs of illness or abnormal behavior prior to the experiment were excluded. Mice were monitored daily for health status. The mice that showed severe distress or reached humane endpoints were euthanized and excluded from the final analysis.

Mice were randomly assigned to control or experimental groups. Adenovirus carrying a Cre-recombinase allele (Ad-Cre) was applied through intratracheal injection for CREPT depletion in lung. Mice were anesthetized and a total of 60 µL aliquot composing Ad-Cre (5x10^7^ PFU) and 10 mM CaCl_2_ in Minimum Essential Medium (MEM) were given. Ad-GFP was used as control. To induce KRAS^G12D^ expression, the mice were provided with drinking water containing 0.15% doxycycline (Dox, g/v) and 5% sucrose. To induce systemic deletion of CREPT in CreERT2+/-;TetO--*KRAS^G12D^*;*CREPT^flox/flox^* mice, tamoxifen was intraperitoneal injected with 100 µL of tamoxifen (2 mg) per day for 5 consecutive days. Tamoxifen was prepared in sunflower seed oil/ethanol mixture (10:1) at 20 mg/mL.

### Micro-CT

Micro-CT (Micro-CT, Quantum GX microCT imaging system) was employed to scan lung tumor formation according to the instruction. Briefly, the mice were anesthetized with isoflurane and placed in the scanning area. The scan was at 90 V using a high-resolution mode for 4 min. Mimics Medical was used for 3D reconstruction of tumors. During analysis the micro-CT results, the mice numbers and groups were blinded until after the analysis were completed.

### Subcutaneous tumor xenografts

H441 and A549 tumor xenografts were established by subcutaneously injecting 1
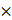
10^6^ to 5
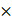
10^6^ cells to 6- to 8-week-old male nude mice. Tumors were harvested at week 4 for H441 cells and week 8 for A549 cells. For CDK9 inhibitor (CDK9i) treatment, H441 cells were resuspended in a 1:1 mixture of HBSS and Matrigel at a final concentration of 5
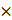
10^7^ cells/mL. Each NU/NU nude mouse was injected with 100 µL of the cell suspension. Tumor volumes were calculated using the empirical formula V = length × width^2^
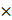
0.52. Once tumor volumes reached approximately 100 mm^3^, mice were treated with either vehicle or 12.5 mg/kg CDK9i (BAY 1251152, also known as VIP152) once weekly. All tumors were collected at week 6 post-injection.

### Establishment of humanized mouse model and treatments

Six- to eight-week-old male NCG mice were used. Tumor formation was established by subcutaneous injection of 2
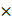
10^6^ H441 cells. For immune reconstitution, peripheral blood mononuclear cells (PBMCs) expressing HLA-A*02:01 were administered intravenously. H441 cell implantation was performed 2 days prior to PBMC introduction. When tumor volumes reached approximately 100 mm^3^ (designated as Day 0), Adeno-associated virus (AAV) serotype 9-mediated 1
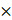
10^11^ vg CREPT shRNAs (AAV-shCREPT) or control shRNAs (AAV-shNC) were administrated. Pembrolizumab was administered at a dose of 10 mg/kg on Days 9, 13, and 17. Tumor dimensions (length and width) and animal body weights were measured weekly throughout the experiment.

### Immunohistochemistry (IHC)-staining

The lung lobes were isolated from mice and fixed in 4% paraformaldehyde at 4 ^o^C overnight. Afterwards, the lungs were embedded in paraffin and sectioned. Human LUAD tissue samples were obtained from patients undergoing surgical resection at The Second Affiliated Hospital of Jiaxing University (Jiaxing, China). The study protocol was approved by the Ethics Committee of The Second Affiliated Hospital of Jiaxing University (approval number: JXSH-2023-259), and written informed consent was obtained from all participants. Human LUAD (HLugA180Su11) and LUSC (HLug-Squ090Lym-01) tissue arrays were obtained from OUTDO BIOTECH (China).

Before performing the IHC-staining, sections were deparaffinized and heated for antigen retrieval. The sections were then stained with hematoxylin and eosin (H&E) or primary antibodies including a polyclonal antibody anti-CREPT (1:100, RRID:AB_2549087, #PA5-31614, Invitrogen) for mouse tissues, anti-CREPT (1:50, 3E10, a monoclonal antibody produced by our lab) for human tissues 32, and anti-KRAS-G12D (1:100, RRID:AB_2728748, #14429, CST) for both human and mouse tissues. After incubated with second antibody, the sections were developed with hematoxylin. The images were obtained using a digital whole-slide scanner (KF-PRO-120, KFBIO) and the software was used for calculating tumor areas.

### Immunofluorescent and multiplex immunofluorescent staining

For immunofluorescent staining of cells, cells were cultured on sterile glass coverslips and fixed with 4% paraformaldehyde. After fixation, cells were permeabilized and 'blocked with bovine serum albumin. The primary antibodies including anti-CDK9 (RRID: AB_2291505, #2316, CST) and anti-CREPT (3E10, produced by our lab) were used followed by several washes to remove unbound antibodies. Subsequently, cells were incubated with a TRICT-anti rabbit and FICT-anti mouse that recognizes the primary antibodies. After washing away excess secondary antibody, cells were counterstained with DAPI to visualize nuclei. Stained cells were visualized under a fluorescence microscope. The multiplex immunofluorescence (multiplex IF) staining of lung tissues from patients and subsequent quantitative analysis were performed using the Leica Biosystems BOND Rx autostainer and analysis platform.

### Real-time quantitative reverse transcription PCR and RNA-seq

Total RNA was extracted by TRIzol (Invitrogen) according to manufacturer's instructions. A total of 2 µg RNA was used for cDNA synthesis. SuperReal PreMix Plus (TIANGEN) was used for RT-qPCR and the experiment was performed using a LightCycler480II Real-time PCR Instrument (Roche). The results were normalized to β-actin and calculated by the 2^-ΔΔCt^ method. For RNA-seq, a total of 2 µg RNA was prepared and sent to BioMarker for library construction, sequencing, and data processing. The primers used are shown in Table S1.

### CCK-8 assay and colony formation

Cell proliferation was measured using a Cell Counting Kit-8 (CCK-8, Beyotime). Cells were seeded into a 96-well plate at a density of 1000 to 3000 cells each well. CCK-8 solution was added at day 0, 1, 3, 5 and 7 of culture and incubated with cells for 2 h to 3 h. The results were observed at 450 nm using a spectrophotometer (FLUOstar Omega). For colony formation assays, cells were seeded into a 6-well plate at a density of 500 to 2000 cells each well according to the cell lines used. Cells were fixed by methanol after 2 to 4 weeks of culture. Colonies were stained using 0.1% crystal violet. The number of colonies was counted by Image J (RRID:SCR_003070).

### Immunoprecipitation

Cells were collected in cell lysis buffer (50 mM Tris-Cl, 150 mM NaCl, 1% Nonidet P-40, 0.5% sodium deoxycholate, and 1% SDS, pH 8.0) with freshly added protease inhibitors. Cell lysates were then subjected to sonication using a Vibra-Cell sonicator (VSC130) for 5 cycles of 30 s each at 40% amplitude to ensure thorough lysis. Anti-CREPT (3E10), anti-Ser2 (RRID:AB_304749, ab5095, abcam), anti-CDK9 (RRID:AB_2291505, #2316, CST), anti-M2 (RRID:AB_262044, #F1804, Sigma), and anti-HA (RRID:AB_2894930, sc-7392, Santa) were used for immunoprecipitation and 30 µL Protein A/G-agarose beads were used depending on the primary antibody. Cell lysate and antibody were mixed and incubated overnight at 4 °C, rotating. Protein A/G beads were added next day and incubated on rotator for 3 h at 4 °C. Then the samples were washed with wash buffer (150 mM NaCl, 50 mM Tris pH 7.5, 0.05% NP-40) with protease inhibitors for 4 times. The immunoprecipitated proteins were then analyzed by adding 30 µL loading buffer (50 mL loading buffer containing 2.5 g SDS, 0.02 g bromophenol blue, 10 mL glycerol, 5 mL 1M Tris-HCl, pH 6.8, and 2.5 mL β-mercaptoethanol) and subjected to Western blot.

For the *in vitro* binding assay, we purified CREPT from E. coli, and HA-CREPT and Flag-CDK9 proteins from HEK-293T cells. The purified proteins were incubated together on rotators at 4 °C overnight. Subsequently, the samples were immunoprecipitated using an anti-HA or anti-CREPT antibodies.

### Luciferase assay

Dual-luciferase reporter experiments were performed in HEK-293T WT and CREPT-knockout (KO) cells using a luciferase reporter of Elk-1, pFA-ELK1/pFR-luc. Briefly, 100 ng of each reporter plasmid and 5 ng of superTop-luc were transiently transfected into cells. HA-CREPT or HA-pcDNA3.1 plasmids were sequentially added. In addition, KRAS^WT^ or a sustainably activated form of KRAS (KRAS^G12D^) were transfected to stimulate cells. After 24 h of transfection, cells were collected, and luciferase activity was measured using a Dual Luciferase reporter assay system (Vigorous Biotechnology) by a microplate reader.

### EGF, FGF stimulation and mitogen release

Cell starvation was performed in serum-free medium for 8 h in HEK-293T cells and 12-24h in SK-LU-1 cells. Then, 100 ng/mL of EGF or 10 ng/mL FGF diluted in serum-free medium were added to stimulate cells. For mitogen release, cell medium containing 20% of FBS was added to cells for 10 to 30 min.

### Chromatin immunoprecipitation (ChIP)

Chromatin Immunoprecipitation (ChIP) was performed. Briefly, 1x10^7^ cells were used for each sample. The cells were fixed in 0.75% formaldehyde for 7 min and quenched by 125 mM glycine. The cell lysate was subjected to sonication using a Vibra-Cell sonicator (VSC130) for 30 cycles of 30 s each at 50% amplitude. This sonication protocol was optimized to obtain DNA fragments ranging from 100 to 500 bp in length. Approximately 25 µg of DNA was used for each sample and 6 µg antibody was added. Anti-CREPT (3E10, produced by our lab), anti-RNAPII (Rpb1-NTD, #14958, CST, USA), and anti-CDK9 (ab239364, Abcam, USA) were employed for immunoprecipitation. The precipitated DNA was tested using RT-qPCR for analysis. The primers used are shown in Table S1.

### Western blot

For western blot, sample were loaded and subjected to 10% sodium dodecyl sulfate polyacrylamide gel electrophoresis (SDS-PAGE). Proteins were transferred to nitrocellulose blotting membranes (GE Healthcare Life Sciences). The primary antibodies used are listed in Table S2. Immunoreactive bands were developed using an enhanced chemiluminescence (ECL) kit (Thermo Fisher Scientific) and visualized using a SAGECREATION system.

### Statistical analysis

RNA-seq results, including Gene Set Enrichment Analysis (GSEA), were analyzed using R software (version 4.4.1, RRID:SCR_001905). GSEA was chosen for its ability to analyze the entire spectrum of gene expression changes, rather than focusing solely on the most significantly differentially expressed genes (DEGs). PRISM 6.0 (GraphPad, RRID:SCR_002798) was utilized for statistical data analysis. The The Cancer Genome Atlas Program (TCGA, RRID:SCR_014514) RNA-seq data were downloaded from Xena platform 33, the analysis for OS and relapse-free survival (RFS) analysis were processed using the KM Plotter tool (http://kmplot.com/analysis/) 34, 35.

All data are presented as mean ± standard deviation (SD). GEPIA (RRID:SCR_018294) was used to analyze Pearson's correlation coefficient (r) to evaluate the correlation 36. Statistical significance was assessed using either unpaired two-tailed Student's t-test for normally distributed data or Mann-Whitney U test for non-normally distributed data. Normality was assessed using the Shapiro-Wilk test. Statistical significance was assessed using the t-test for normal distribution or Mann-Whitney U test. A p-value of less than 0.05 was considered significant. *, P < 0.05; **, P < 0.01; ***, P < 0.001; ****, P < 0.0001.

## Results

### CREPT is highly expressed in LUAD and correlates with its recurrence

**Figure 1:**
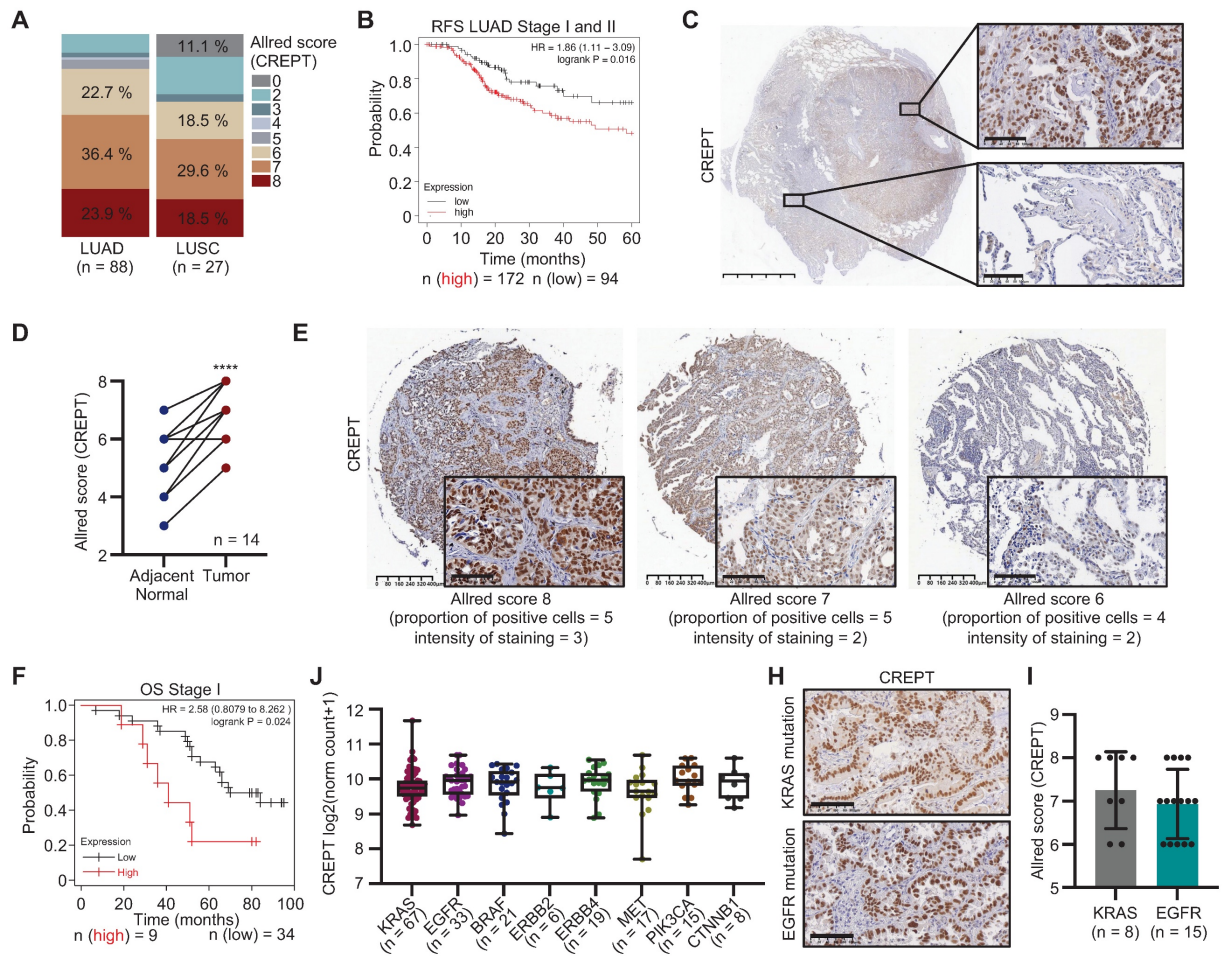
** CREPT expression in lung adenocarcinoma (LUAD) and its correlation with patient outcomes.** (A) The quantitative analysis of CREPT expression levels (Allred scores) in LUAD (n = 88) and LUSC (n = 27) patient samples using immunohistochemistry (IHC) staining of two tissue arrays. (B) Relapse-free survival (RFS) analysis of stage I and II LUAD patients based on CREPT expression levels (high vs. low, n = 172 and n = 94, respectively). (C) Representative IHC images of CREPT expression in LUAD tissue. Scale bar: 5 mm and 100 µm. (D) Paired analysis of CREPT expression (Allred scores) in adjacent normal and tumor tissues from LUAD patients (n = 14). (E) Representative IHC images showing different CREPT expression levels in LUAD tissues of a tissue array with survival information. Allred scores and staining characteristics are indicated. Scale bars: 400 μm and 100 μm. (F) Overall survival (OS) analysis of stage I LUAD patients according to the CREPT expression level in the LUAD tissue array (high vs. low, n = 9 and n = 34, respectively). (J) CREPT mRNA expression levels across different LUAD subtypes based on genetic alterations using RNA-seq analysis in TCGA database. (H) Representative IHC images of CREPT expression in KRAS-mutant and EGFR-mutant tumor tissues of LUAD patients. (I) Comparison of CREPT expression (Allred scores) between KRAS-mutant (n = 8) and EGFR-mutant (n = 15) LUAD samples. Data are presented as mean ± SD. Statistical significance was determined by paired t-test for expression comparisons. ****p < 0.0001.

To elucidate the role of CREPT in NSCLC, we examined its expression and association with the clinicopathological features in LUAD and LUSC. Analysis of the TCGA lung cancer cohort revealed significantly elevated CREPT mRNA levels in various subtypes of LUAD and LUSC compared to adjacent normal tissues (Figure S1A-B). IHC staining of tissue arrays confirmed higher CREPT protein expression in tumor tissues of both LUAD and LUSC relative to paired adjacent tissues (Figure S1C-E). Quantification using the Allred scoring system demonstrated increased CREPT protein expression, with higher levels in LUAD compared to LUSC (Figure 1A). Further analyses indicated that elevated CREPT expression correlated with decreased OS and RFS in early-stage patients in LUAD (Stages I and II, Figure 1B; Figure S1F) but not in LUSC (Figure S1G). These findings highlight the potential significance of CREPT in LUAD progression and recurrence, prompting us to focus on its role specifically in LUAD.

To validate CREPT expression in LUAD, we analyzed 14 tumor samples from LUAD patients. IHC analyses confirmed significantly higher CREPT protein levels in tumor tissues compared to adjacent normal tissues (Figure 1C-D). Consistently, CREPT protein expression was substantially higher in several LUAD cell lines compared to fibroblasts and non-tumorigenic epithelial cells (Figure S1H). To assess the correlation between CREPT protein level and clinical outcome, we performed IHC staining on a tissue array of LUAD patients with survival information, categorizing patients into high (Allred score = 8) and low (Allred score < 8) CREPT expression groups (Figure 1E). Kaplan-Meier analysis revealed significantly poorer OS in early-stage patients with higher CREPT expression (n = 90, Figure 1F; Figure S1I), corroborating our RNA-seq data analysis from TCGA (Figure 1B; Figure S1F).

Further analyses using the TCGA database demonstrated significantly higher CREPT levels in stage IV patients and those with metastasis compared to early-stage patients and those with primary tumors, while no difference was observed in other patient demographics or genetic mutations (Table S3, Figure 1J-I; Figure S1J). Additionally, analysis of TCGA data using the Xena platform revealed a correlation between elevated CREPT mRNA levels and increased CREPT gene copy number in LUAD (Figure S1K). Collectively, these findings indicate that CREPT is highly expressed in LUAD and correlates with OS, metastasis, and recurrence, suggesting its crucial role in disease pathogenesis and progression.

### CREPT promotes proliferation and tumorigenesis of LUAD cells

**Figure 2:**
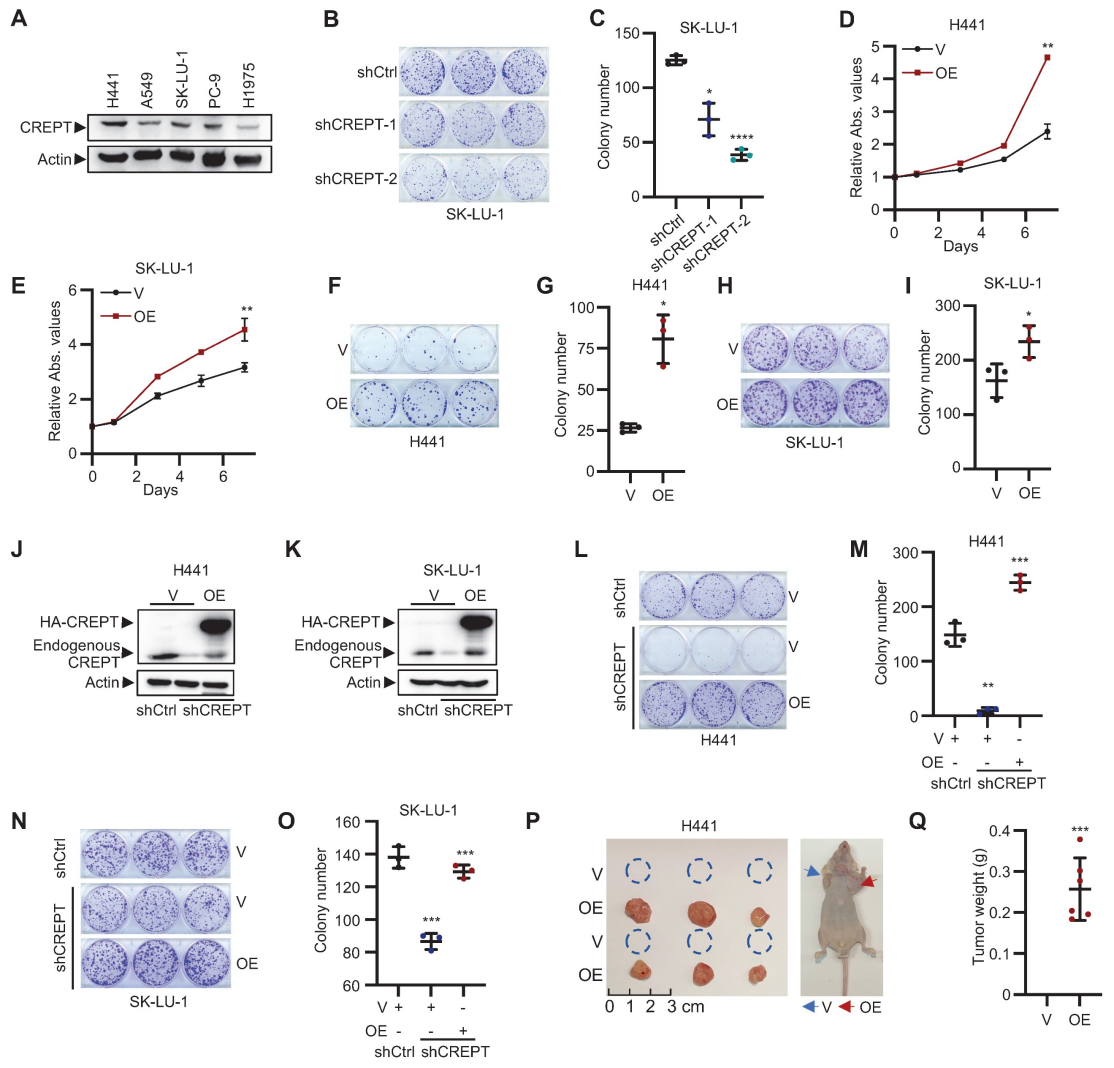
** CREPT promotes proliferation and tumorigenesis of LUAD cells.** (A) Western blot analysis of CREPT expression in various LUAD cell lines harboring *KRAS* or* EGFR* mutants. (B-C) Colony formation assay of SK-LU-1 cells with CREPT knockdown. Representative images (B) and quantification (C). (D-E) Cell Counting Kit-8 (CCK-8) assays of CREPT-overexpressing (OE) vs. control (V) H441 (D) and SK-LU-1 (E) cells. (F-I) Colony formation assays in CREPT-OE vs. control (V) H441 (F-G) and SK-LU-1 (H-I) cells. Representative images (F, H) and quantification (G, I). (J-K) Western blot analysis of CREPT restoration levels in CREPT knockdown cells of H441 (J) and SK-LU-1 (K). (L-O) Colony formation assay in CREPT-knockdown and rescue H441 (L-M) and SK-LU-1 (N-O) cells. Representative images (L, N) and quantification (M, O). (P) Subcutaneous tumor growth of H441 CREPT-OE vs control (V) in nude mice. Representative images of tumors (left) and mice (right). (Q) Quantification of tumor weights from the experiment in (P). Data are presented as mean ± SD from of three biological replicates. Statistical significance was determined by two-tailed Student's t-test. *p < 0.05, **p < 0.01, ***p < 0.001, ****p < 0.0001.

To elucidate the functional role of CREPT in LUAD, we examined its impact on cell proliferation in five LUAD cell lines with *KRAS* or* EGFR* mutations and a gefitinib resistant cell line. Initial analysis revealed high CREPT protein expression in H441 and SK-LU-1 cells (Figure 2A). CREPT depletion *via* shRNA, validated by RT-qPCR and western blot (Figure S2A-B), significantly inhibited cell proliferation and colony formation in these cell lines (Figure 2B-C; Figure S2C-E). Transient CREPT silencing using two siRNAs markedly decreased colony formation in H441 cells (Figure S2F-H). Furthermore, CREPT depletion also suppressed clonogenicity in A549, PC-9, PC-9/GR, H1975 cells (Figure S2I-N). This effect was consistent across KRAS-independent (SK-LU-1, A549^37^) and gefitinib-resistant (PC-9/GR) lines.

Reciprocally, CREPT overexpression in H441, SK-LU-1 and A549 cells markedly enhanced cell proliferation and increased colony formation (Figure 2D-I; Figure S2O-R). Rescue experiments, re-introducing CREPT in stable knockdown lines of H441 and SK-LU-1 (Figure 2J-K), substantially restored colony formation capacity (Figure 2L-O). These results collectively demonstrate the crucial role of CREPT in promoting LUAD cell proliferation and colony formation, irrespective of *KRAS* or* EGFR* mutational status or gefitinib resistance.

To validate our *in vitro* findings, we employed xenograft models using nude mice. LUAD cells overexpressing CREPT were subcutaneously injected to assess the impact of CREPT on tumorigenesis. Strikingly, CREPT-overexpressing H441 cells exhibited a pronounced ability to form tumors, while the control cells form no tumors at the same time point (Figure 2P-Q). This effect was corroborated in A549 cells, where CREPT overexpression resulted in significantly larger and heavier tumors compared to controls (Figure S2S). Taken together, these findings demonstrate that CREPT significantly contributes to LUAD cell proliferation, colony formation, and tumorigenesis.

### CREPT depletion downregulates the KRAS-ERK signaling

**Figure 3:**
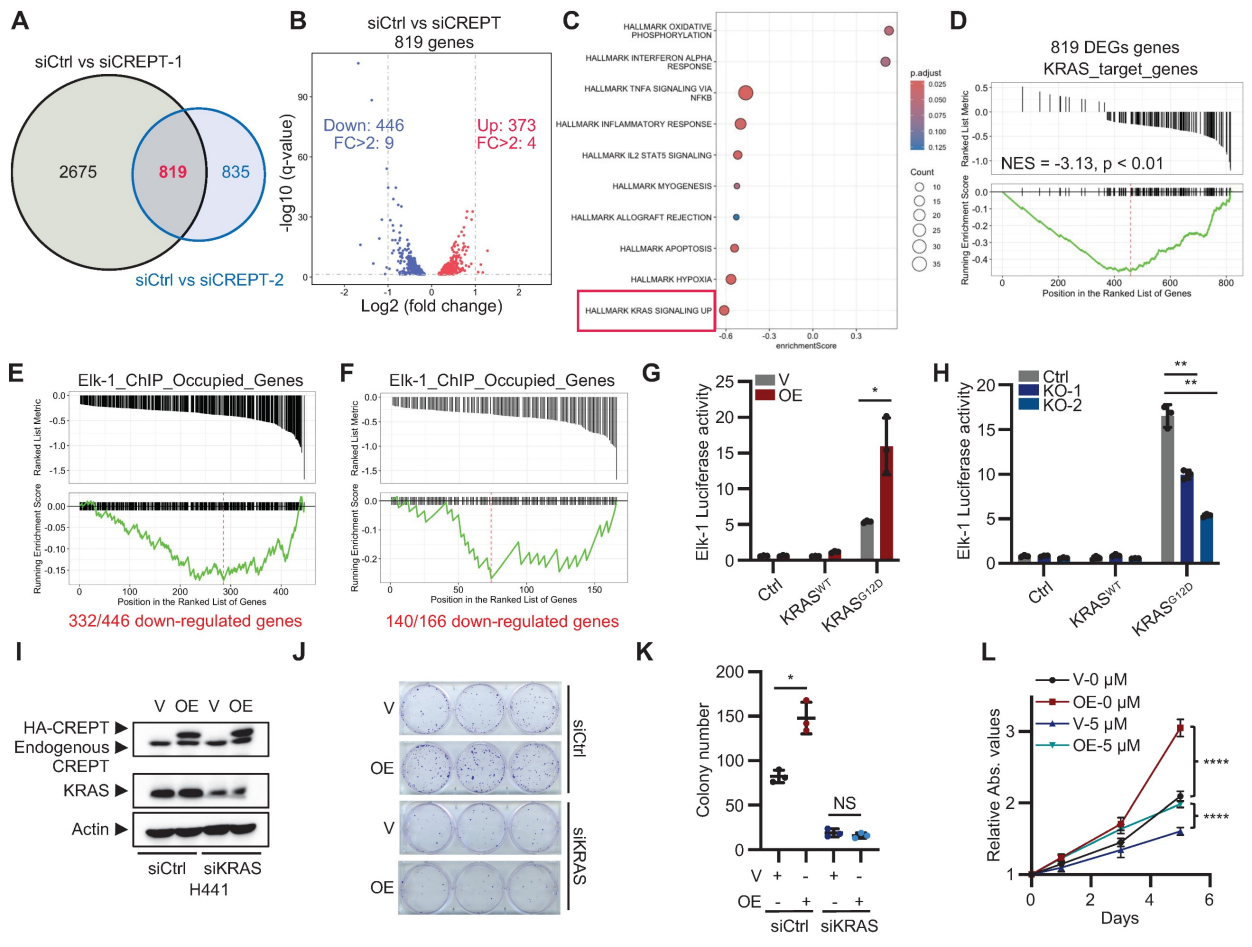
** CREPT regulates the expression of KRAS-ERK-Elk-1 downstream genes.** (A) Venn analysis showing overlapping differentially expressed genes (DEGs) between siCtrl vs siCREPT-1 and siCtrl vs siCREPT-2 in H441 cells. (B) Volcano plot of 819 common DEGs between siCtrl and siCREPT conditions (A new gene without name was deleted). Red: upregulated; Blue: downregulated. (C) Gene set enrichment analysis (GSEA) of DEGs, highlighting enrichment of hallmark gene sets. (D) GSEA plot showing enrichment of KRAS target genes among the 819 DEGs in H441 cells. (E-F) GSEA-style enrichment plots showing enrichment of Elk-1 ChIP-occupied genes among downregulated genes in siCREPT conditions: 466 genes in H441 cells (E) and 166 genes common to both H441 and SK-LU-1 cells (F). (G) Elk-1 luciferase reporter assay in HEK-293T cells with vector (V) or CREPT overexpression (OE) stimulated using vector (V), wild-type (WT) or constitutively active KRAS (KRAS^G12D^). (H) Elk-1 luciferase reporter assay in HEK-293T WT and CREPT knockout (KO) cells, stimulated using vector (V), wild-type KRAS (WT), or KRAS^G12D^ mutant. (I) Western blot analysis of CREPT and KRAS expression in H441 cells transfected with indicated siRNAs. (J-K) Colony formation assay of H441 cells with CREPT overexpression (OE) and/or KRAS knockdown (siKRAS). Representative images (J) and quantification (K). (L) CCK-8 assay of H441 cells with CREPT overexpression (OE) treated with the ERKi (5 µM). Data are presented as mean ± SD from of three biological replicates. Statistical significance was determined by two-tailed Student's t-test. *p < 0.05, **p < 0.01, ****p < 0.0001, NS: not significant.

To elucidate the signaling pathways that CREPT participates in LUAD, we performed RNA-seq analysis on H441 cells following CREPT depletion using two specific siRNAs. We identified 819 consistently altered genes by both siRNAs, with 446 downregulated and 373 upregulated (Figure 3A-B; Figure S3A; Table S4). GSEA revealed a negative correlation between the DEGs and the KRAS signaling gene set (Figure 3C), suggesting the involvement of CREPT in KRAS signaling. To validate this finding, we generated KRAS-depleted H441 cells (Figure S3B-C) and identified 897 downregulated genes using RNA-seq (Table S5), which we designated as KRAS-regulated genes. We then used this set of KRAS-regulated genes as a reference gene set to perform GSEA on the 819 DEGs resulting from CREPT depletion. This analysis showed a significant negative correlation between CREPT-regulated and KRAS-regulated genes (NES = -3.13, p < 0.01, Figure 3D), further supporting the role of CREPT in KRAS signaling regulation.

Given that CREPT is a nuclear-localized protein that directly interacts with RNAPII 25, we hypothesized that it might function as a co-factor in activating ERK downstream genes. To investigate this, we examined whether CREPT-regulated genes were occupied by Elk-1, a key transcription factor downstream of ERK in LUAD development. Using ChIP-seq data from the ENCODE Transcription Factor Targets Dataset, we created an Elk-1_ChIP_Occupied_Genes set. Venn analysis revealed that 332 out of 446 CREPT-downregulated genes in H441 cells were occupied by Elk-1 (Figure S3D). We visualized these 332 genes using a GSEA-style enrichment plot, demonstrating their distribution within the Elk-1_ChIP_Occupied_Genes set (Figure 3E). Similar analysis in SK-LU-1 cells revealed 140 out of 166 consistently downregulated genes were Elk-1 occupied (Figure 3F; Figure S3E-H), supporting that CREPT may modulate KRAS signaling through cooperation with Elk-1.

To further investigate the impact of CREPT on the Elk-1 function, we employed Elk-1 luciferase reporter assays. CREPT overexpression significantly enhanced, while its depletion diminished, Elk-1 transcriptional activity in response to KRAS^G12D^ stimulation, EGF, and FGF stimulation (Figure 3G-H; Figure S3I). Notably, CREPT did not significantly affect the phosphorylation status or total expression levels of other KRAS signaling components, including KRAS, ERK, RSK, c-Jun, c-Fos, MYC, and Elk-1 (Figure S3J-K), suggesting that CREPT mainly modulate KRAS downstream gene transcription but not activation of the signaling cascade.

To further confirm the function of CREPT in KRAS signaling, we depleted KRAS using siRNAs in CREPT-overexpressing H441 cells. CREPT overexpression failed to promote cell growth or colony formation in KRAS-depleted cells (Figure 3I-K; Figure S3L). Moreover, ERK inhibition (ERKi, SCH772984) markedly suppressed H441 cell proliferation and attenuated the growth-promoting effect of CREPT overexpression (Figure 3L; Figure S3M).

Collectively, these findings demonstrate that CREPT exerts its pro-tumorigenic role through the KRAS-ERK pathway, specifically by enhancing Elk-1 transcriptional activity without altering the activation or expression of upstream signaling components.

### CREPT is required during the initiation of KRAS^G12D^-driven LUAD

**Figure 4:**
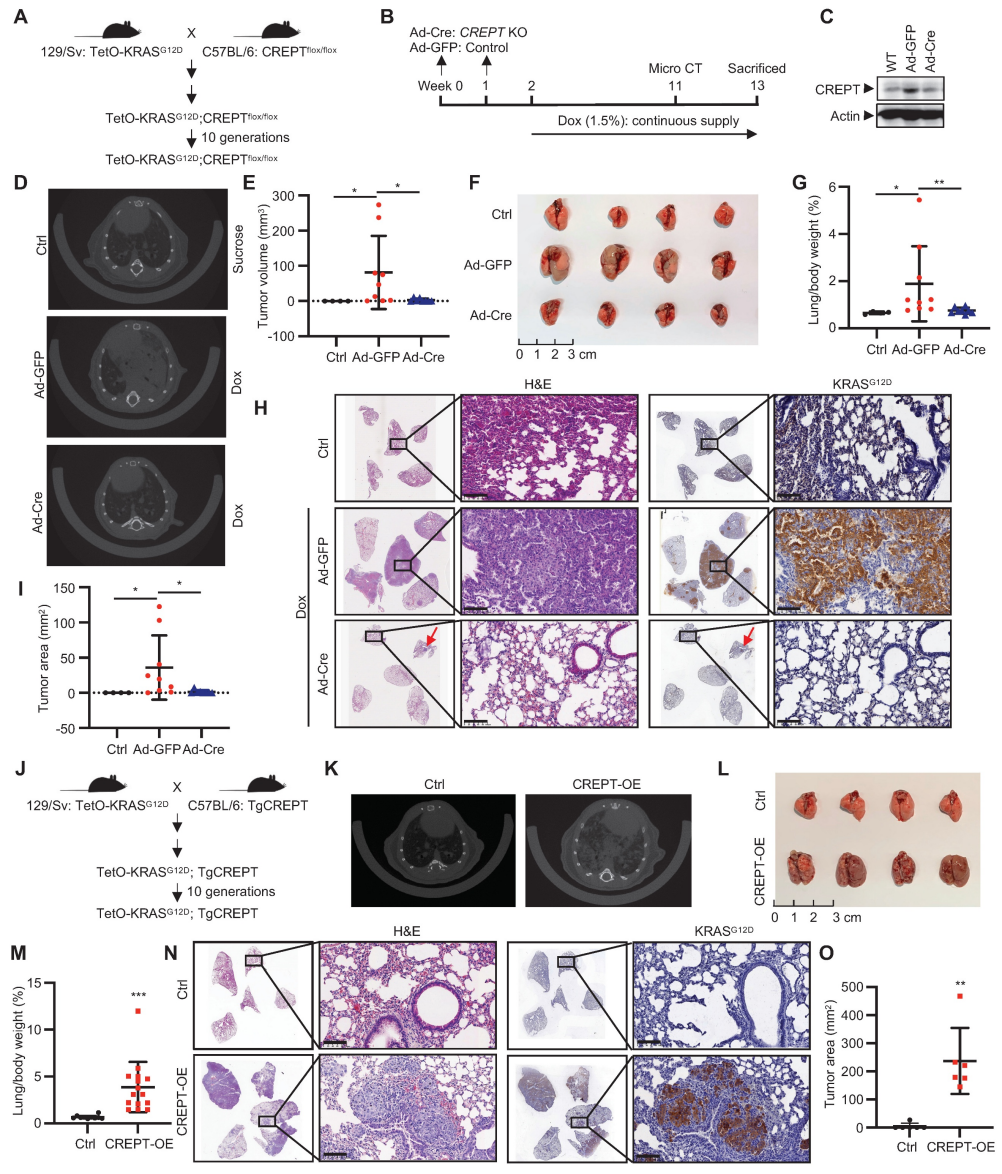
** CREPT promotes KRAS^G12D^-driven lung adenocarcinoma.** (A) Establishment of TetO-*KRAS^G12D^*;*CREPT^flox/flox^* mouse models. (B) Schematic diagram showing the intratracheal injection of Ad-Cre for *CREPT* deletion and doxycycline (Dox) treatment for induction of KRAS^G12D^ expression. (C) Western blot analysis of CREPT expression after Ad-Cre or Ad-GFP intratracheal injection. (D) Representative micro-CT images of lung adenocarcinoma in control, Ad-GFP, and Ad-Cre treated mice. (E) Quantification of tumor volume from micro-CT analysis using mimics (n = 4 for control group, n = 9 for mice treated with Ad-GFP, n = 8 for mice treated with Ad-Cre). (F) Lung tissues of mice in each group at endpoint. (G) Quantification of lung/body weight ratio. (H) H&E staining of lung tissue sections from control, Ad-GFP, and Ad-Cre treated mice. Scale bars: 100 μm. Red arrow points the small tumor observed in Ad-Cre treated mice. (I) Quantification of tumor areas from H&E and KRAS^G12D^ stained sections. (J) Establishment of TetO-*KRAS^G12D^*;TgCREPT mouse models. (K) Representative micro-CT images of lung adenocarcinoma in control (Ctrl) and CREPT-overexpression (CREPT-OE) mice. (L) Lung tissues of mice in each group at endpoint. (M) Quantification of lung/body weight in control and CREPT-OE mice (n = 10 for control group, n = 15 for CREPT-OE group). (N) H&E staining and KRAS IHC of lung tissue sections from control and CREPT-OE mice. Scale bars: 100 μm. (O) Quantification of tumor area from H&E and KRAS^G12D^ stained sections (n = 6 for control group, n = 6 for CREPT-OE group). Data are presented as mean ± SD. Statistical significance was determined by two-tailed Student's t-test. *p < 0.05, **p < 0.01, ***p < 0.001, NS: not significant.

To investigate the role of CREPT in KRAS-driven LUAD, we generated a TetO-*KRAS^G12D^*;*CREPT^flox/flox^* mouse line to induce KRAS^G12D^ expression and CREPT depletion (Figure 4A). CREPT depletion was achieved *via* intratracheal injections of Ad-Cre, while KRAS^G12D^ expression was induced by Dox administration (Figure 4B). Adenoviral infection efficiency was validated through GFP immunostaining of lung tissues (Figure S4A). Western blot analysis confirmed CREPT depletion in lung tissues (Figure 4C). After 11 weeks, micro-CT examination revealed adenocarcinoma in 88.9% (8/9) of Ad-GFP-treated mice compared to 62.5% (5/8) in Ad-Cre-treated mice (Figure 4D; Figure S4B). Quantitative analysis showed significantly reduced tumor volumes in Ad-Cre-treated mice relative to Ad-GFP controls, which exhibited substantially increased tumor volumes (Figure 4E). Of note, large tumors (tumor volume > 20 mm^3^) were observed in 5/9 Ad-GFP-treated mice but none in Ad-Cre-treated mice (Figure S4B).

To further assess the progression of tumor development, we sacrificed all mice at Week 13 (Figure 4B). Ad-GFP-treated mice developed enlarged tumors in their lungs, while Ad-Cre-treated mice showed minimal tumor growth (Figure 4F; Figure S4C). Quantitative analysis revealed a decreased lung/body weight ratio in CREPT-deleted mice (Figure 4G). H&E analyses of Ad-GFP-treated mice revealed morphological features characteristic of adenocarcinoma, including significant cellular atypia, disorganized tissue structure with disrupted glandular architecture, and poor overall differentiation. IHC confirmed positive KRAS^G12D^ staining in these tumor areas. In contrast, Ad-Cre-treated mice exhibited only small tumors in both H&E and IHC analyses (Figure 4H). CREPT staining was strong in Dox-induced tumors but weak in lungs from Ad-Cre-treated mice (Figure S4D). Quantitative analysis across all five lobes showed significantly reduced total tumor area in Ad-Cre-treated mice compared to Ad-GFP controls (Figure 4I). We occasionally observed scattered tumors in Ad-Cre-treated mice lungs (Figure S4E), which stained positively for both KRAS^G12D^ and CREPT (Figure S4E, enlarged box). We attribute these to incomplete *CREPT* deletion, likely due to the imperfect efficiency of intratracheal Ad-Cre instillation. Collectively, these results strongly suggest that *CREPT* deletion impairs KRAS^G12D^-induced lung adenocarcinoma formation.

To further elucidate the functions of CREPT in regulating KRAS-driven tumorigenesis, we generated a transgenic mouse (Tg-*CREPT*) and crossed with EIIα-Cre to overexpress CREPT (Figure S4F-G). In general, we observed that this mouse developed normally under routine feeding conditions (data not shown). We then crossed this mouse line with TetO-*KRAS^G12D^* mice to generate a mouse model that allows for induction of KRAS^G12D^ expression with CREPT overexpression (denoted as Tg-*CREPT*;TetO-*KRAS^G12D^*, Figure 4J). Following Dox-induced KRAS^G12D^ expression, micro-CT imaging at Week 3 revealed dramatic adenocarcinoma formation in CREPT-overexpressing mice, while control mice rarely developed adenocarcinoma (Figure 4K). All mice overexpressing CREPT developed large tumors (15/15), while only one control mouse exhibited tumor (1/10) (Figure 4L-M). CREPT-overexpressing mice exhibited significantly higher lung/body weight ratios (Figure 4M). H&E and IHC analyses confirmed large KRAS^G12D^-expressing tumors in CREPT-overexpressing mice (Figure 4N). Quantitative analysis showed that these tumors were significantly larger in area compared to those in control mice (Figure 4O). IHC analysis confirmed the overexpression of CREPT in Tg-*CREPT*;TetO-*KRAS^G12D^* mice relative to TetO-*KRAS^G12D^* mice (Figure S4H). Importantly, CREPT overexpression did not affect KRAS^G12D^ expression levels in tumor areas (Figure S4I).

Collectively, these results provide compelling evidence that *CREPT* deletion impairs, while its overexpression significantly promotes, KRAS^G12D^-induced lung adenocarcinoma formation. These findings underscore the pivotal role of CREPT in enhancing KRAS^G12D^-driven tumorigenesis and suggest its involvement in modulating KRAS-driven oncogenic pathways.

### The cooperative role of CREPT and CDK9 in ERK signaling regulation

To identify CREPT-interacting proteins potentially regulating KRAS-ERK-Elk-1 signaling, we analyzed our previously immunoprecipitation coupled with mass spectrometry (IP-MS) data from HEK-293T cells 38. CDK9, a critical transcriptional regulator, emerged as a strong CREPT-associated partner. Notably, while CREPT enhanced Elk-1 transcriptional activity, no physical interaction was observed between CREPT and Elk-1 proteins (Figure S5A).

**Figure 5:**
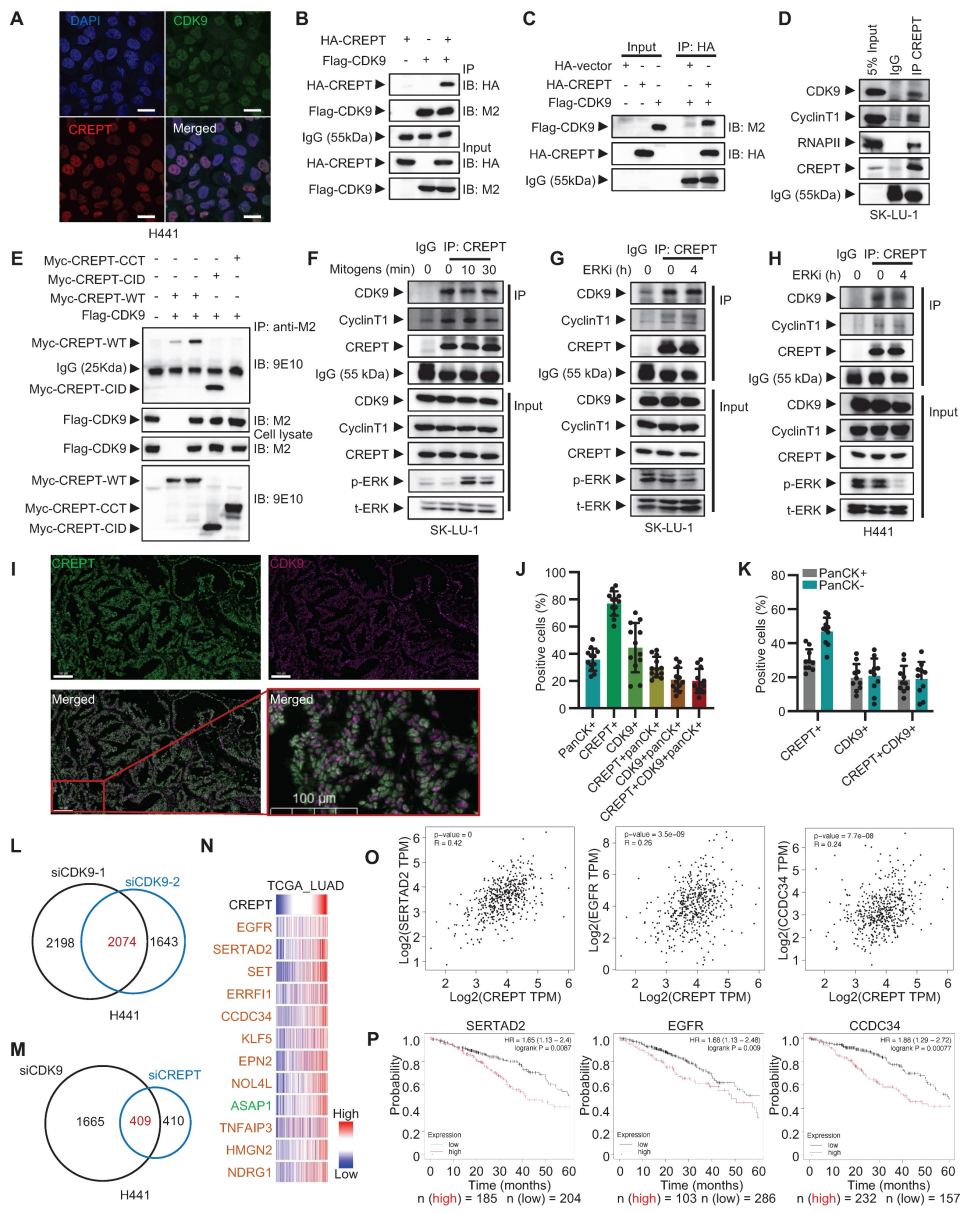
** The cooperative role of CREPT and CDK9 in LUAD.** (A) Immunofluorescence images showing nucleus localization of CDK9 (green) and CREPT (red) in H441 cells. Nuclei stained with DAPI (blue). Scale bar: 20 μm. (B) HEK-293T cells were co-transfected with plasmids expressing HA-CREPT and Flag-CDK9. Co-immunoprecipitation assay was performed using an anti-Flag antibody (M2), and samples were analyzed by Western blotting with anti-HA and anti-FLAG antibodies to detect CREPT and CDK9, respectively. (C) Flag-CDK9 and HA-CREPT proteins were individually purified from HEK-293T cells and the purified proteins were incubated together at 4 °C overnight. Immunoprecipitation assay was performed using an anti-HA antibody, and Western blotting with anti-HA and anti-Flag antibodies to detect CREPT and CDK9, respectively. (D) The endogenous interaction between CREPT and CDK9 in SK-LU-1 cells. (E) An immunoprecipitation assay of Myc-tagged CREPT and its two domains CID and CCT with Flag-CDK9. Western blotting with anti-Myc (9E10) and anti-Flag (M2) antibodies to detect CREPT and CDK9, respectively. (F-H) Immunoprecipitation assays showing the interactions between CREPT and CDK9/Cyclin T1 in response to mitogen stimulation or ERK inhibition in SK-LU-1 and H441 cells. (I) The multiplex immunofluorescence (IF) staining of lung tissues from patients using the Leica Biosystems, Bond RX platform. Scale bar: 100 μm. (J) Quantitative analysis of multiplex IF staining for CREPT, CDK9 and pan-cytokeratin (panCK) positive cells (n = 12). (K) Quantitative analysis of multiplex IF staining for CREPT, CDK9 positive cells in panCK negative or positive cells. (L) Venn analysis showing overlap of genes regulated by siCDK9-1 and siCDK9-2. (M) Venn analysis illustrating the overlap of DEGs in H441 cells treated with siCDK9 or siCREPT. (N) Heatmap showing the correlation of CREPT and the genes co-downregulated by CDK9 and CREPT using RNA-seq from TCGA database. (O) The correlations between *CREPT*, *EGFR*, *SERTAD2*, and *CCDC34* expression analyzed by GEPIA in LUAD tumor tissues. (P) Kaplan-Meier survival curves for LUAD patients based on expression levels of SERTAD2, EGFR, and CCDC34 using Kaplan-Meier Plotter. Data are presented as mean ± SD.

We confirmed the CREPT-CDK9 association through immunofluorescence staining and reciprocal immunoprecipitation experiments. Both CREPT and CDK9 localized to the nucleus in H441 cells (Figure 5A). Immunoprecipitation experiments demonstrated interaction between exogenously expressed HA-CREPT and Flag-CDK9 in HEK-293T cells (Figure 5B). Furthermore, *in vitro* binding assays using purified proteins showed direct interaction between CREPT and CDK9 (Figure 5C; Figure S5B). This was observed both when HA-CREPT was purified from HEK-293T cells and when CREPT was purified from E. coli, with Flag-CDK9 purified from HEK-293T cells. Endogenous interactions between CREPT and CDK9 were observed in SK-LU-1 and H441 cells (Figure 5D; Figure S5C). Domain mapping analysis revealed that the CID domain of CREPT mediated its interaction with CDK9 (Figure 5E). Interestingly, the CREPT-CDK9 interaction remained constant under mitogen stimulation (Figure 5F) and ERK inhibition conditions (Figure 5G-H; Figure S5D), suggesting independence of CREPT-CDK9 formation from ERK signaling.

To investigate the clinical relevance of the CREPT-CDK9 interaction, we performed multiplex IF experiments on LUAD patient samples harboring *KRAS* or* EGFR* mutations (Figure 5I). Quantitative analysis (n = 10) revealed that pan-cytokeratin-positive (panCK+) tumor cells accounted for approximately 35% of total cells, with CREPT-positive (CREPT+) and CDK9-positive (CDK9+) cells comprising 77% and 40 %, respectively (Figure 5J). Notably, CREPT and panCK double positive cells accounted for 30% of the total cells. Over 80% (30/35) of panCK+ tumor cells expressed CREPT while 57% expressed CDK9. On the other hand, we found that CDK9, CREPT, and panCK triple positive cells constituted about 19% in tumor tissues while over 90% of CDK9+panCK+ double positive cells expressed CREPT. Interestingly, we observed that both CREPT and CDK9 were expressed not only in panCK+ tumor cells but also in other cell types within the tumor microenvironment (Figure 5K), suggesting that CREPT and CDK9 may play broader roles beyond tumor cells. Collectively, these analyses demonstrate that CREPT is expressed in most CDK9-positive tumor cells, implying a potential functional interaction or a cooperative role between CREPT and CDK9 in the LUAD tumors.

The high co-localization and the interaction of CREPT-CDK9 in tumor cells prompted us to investigate CDK9 expression in LUAD. Analysis of TCGA RNA-seq data revealed a significant positive correlation between CREPT and CDK9 mRNA levels in LUAD patients (Figure S5E). However, CDK9 expression showed only a modest increase in tumor tissues compared to adjacent non-tumor tissues (Figure S5F). Similarly, the expression of CCNT1, the partner of CDK9, showed no significant difference between tumor and adjacent tissues (Figure S5G). Furthermore, neither CDK9 nor CCNT1 expression levels correlated with OS in stage I and II LUAD patients (Figure S5H-I). These findings suggest that despite strong CREPT-CDK9 co-localization and correlated expression in LUAD, CDK9 is only marginally overexpressed. We speculated that CREPT might serve as a compensatory mechanism to enhance CDK9 activity without significant CDK9 amplification.

To test this hypothesis and elucidate whether CREPT modulates CDK9-mediated transcriptional regulation, we performed RNA-seq analysis on CDK9-depleted H441 cells, identifying 2074 DEGs (Figure 5L; Figure S5J-K). Comparison with CREPT-depleted cells revealed 409 genes mutually regulated by both CREPT and CDK9 (Figure 5M). Validation in SK-LU-1 cells confirmed 193 consistently regulated genes, with 69 showing downregulation (Figure S5L, Table S6). Integration of our RNA-seq data with TCGA database for LUAD patients showed that expression levels of the top 12 DEGs significantly correlated with CREPT expression in LUAD tumor tissues (Figure 5N-O; Figure S5M). High expression of SERTAD2, EGFR, CCDC34, and ERRFI1 was associated with poor prognosis in LUAD patients (Figure 5P; Figure S5N), indicating that CREPT and CDK9 may collaborate to enhance expression of malignancy-associated genes. To further investigate the relationship between these genes and the KRAS-ERK pathway, we performed Venn analysis using the aforementioned Elk-1_ChIP_Occupied_Genes set. We found that 60 out of 69 downregulated genes were occupied by Elk-1 (Figure S5O). The 60 genes were visualized using a GSEA-style enrichment plot (Figure S5P). Taken together, these findings demonstrate that CREPT interacts with CDK9 to regulate a set of Elk-1 occupied genes, potentially contributing to LUAD progression and poor patient outcomes.

### CREPT facilitates CDK9-RNAPII interaction and enhances CDK9 occupancy at Elk-1 targets

**Figure 6:**
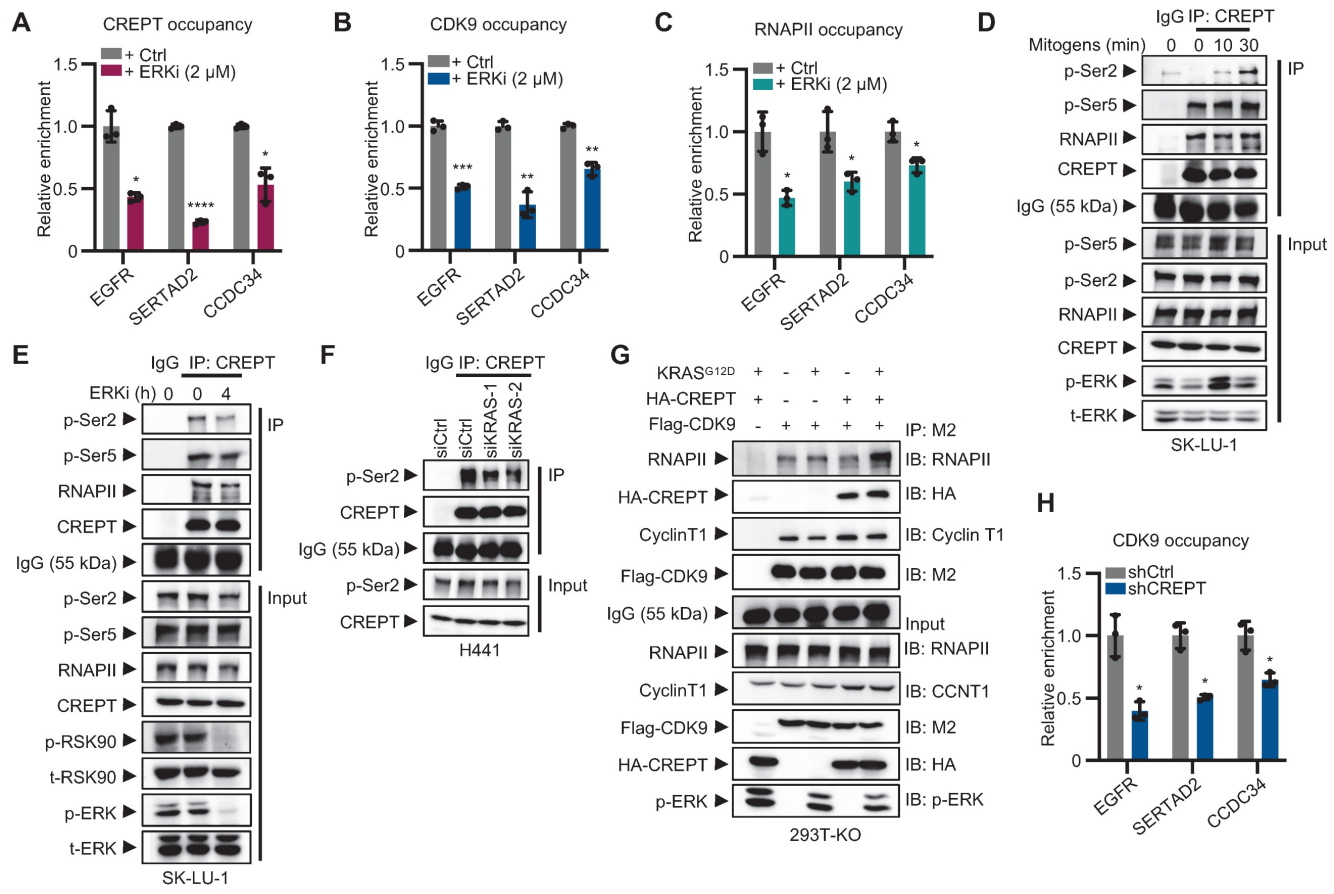
** CREPT mediates CDK9-RNAPII interaction and CDK9 recruitment to Elk-1 occupied genes in LUAD.** (A-C) The occupancy of CREPT (A), CDK9 (B), and RNA polymerase II (RNAPII) (C) on *EGFR*, *SERTAD2*, and *CCDC34* promoters with and without ERKi (2 µM). (D-E) Co-immunoprecipitation of CREPT with RNAPII, p-Ser2 and p-Ser5 in SK-LU-1 cells after ERKi treatment (D) or mitogen release (E). (F) Co-immunoprecipitation of CREPT and p-Ser2 in H441 cells under siCtrl or siKRAS conditions. (G) Co-immunoprecipitation analysis showing the interaction between Flag-CDK9 and RNAPII in CREPT KO cells transfected with CREPT or/and KRAS^G12D^. (H) ChIP-qPCR analysis of CDK9 occupancy at the promoters of *EGFR*, *SERTAD2*, and *CCDC34* in H441 cells with or without CREPT depletion. Data are presented as mean ± SD from of three biological replicates. Statistical significance was determined by two-tailed Student's t-test. *p < 0.05, **p < 0.01, ***p < 0.001, ****p < 0.0001.

To investigate whether CREPT and CDK9 respond to ERK signaling activation, we performed ChIP-qPCR to examine their occupancy on the promoters of Elk-1 downstream genes following ERK inhibition. The results showed that ERKi significantly reduced the occupancies of CREPT, CDK9 and RNAPII on Elk-1 target genes, including EGFR, SERTAD2 and CCDC34 (Figure 6A-C; Figure S6A-B). These findings suggest that CREPT, CDK9 and RNAPII are recruited to Elk-1 downstream genes upon ERK activation.

While the CREPT-CDK9 interaction remains unaffected by ERK activation (see Figure 5F-H), we found that ERK signaling affect the interaction between CREPT and RNAPII. The association between CREPT and RNAPII, particularly with the phosphorylated Ser2 (p-Ser2), but not Ser5 (p-Ser5), was enhanced by mitogen stimulation and inhibited by the ERKi (Figure 6D-F; Figure S6C-D). This indicates that ERK signaling activation enhances CREPT-RNAPII interaction. We further investigated CDK9-RNAPII interaction in the presence or absence of CREPT. KRAS^G12D^ stimulation did not enhance CDK9-RNAPII binding without CREPT (Figure 6G; line 2 vs line 3). However, CREPT overexpression induced an enhanced interaction between CDK9 and RNAPII after KRAS^G12D^ stimulation (Figure 6G; line 3 vs line 5), suggesting that CREPT mediates CDK9-RNAPII interaction. ChIP-qPCR analysis confirmed that CREPT depletion decreased CDK9 recruitment to* EGFR*, *SERTAD2*, and *CCDC34* promoters (Figure 6H).

We next examined the phosphorylation status of RNAPII by overexpressing CDK9 in presence or absence of CREPT. In CREPT-depleted cells, CDK9 overexpression did not alter the phosphorylation levels of Ser2 (p-Ser2) on RNAPII, with or without KRAS^G12D^ stimulation (Figure S6E; lane 4 vs line 1; line 8 vs line 5). However, when CREPT was restored, p-Ser2 levels increased with CDK9 overexpression in a dose-dependent manner (Figure S6E; lane 12 vs line 9). These observations suggest that CREPT mediates CDK9-dependent phosphorylation of RNAPII at Ser2.

Collectively, these findings reveal a novel mechanism whereby CREPT mediates ERK signaling-induced transcriptional activation by facilitating CDK9-RNAPII interaction and CDK9 recruitment to Elk-1 target genes in LUAD.

### ERK-mediated phosphorylation of CREPT at S134 facilitates CDK9 recruitment to Elk-1 target genes

To elucidate the mechanism by which ERK signaling enhances CREPT-RNAPII interaction, we investigated CREPT phosphorylation upon KRAS-ERK activation. Initial analysis using an anti-phospho-S/Y/T antibody revealed EGF-induced CREPT phosphorylation (Figure S7A). We hypothesized that ERK might directly interact with and phosphorylate CREPT.

**Figure 7:**
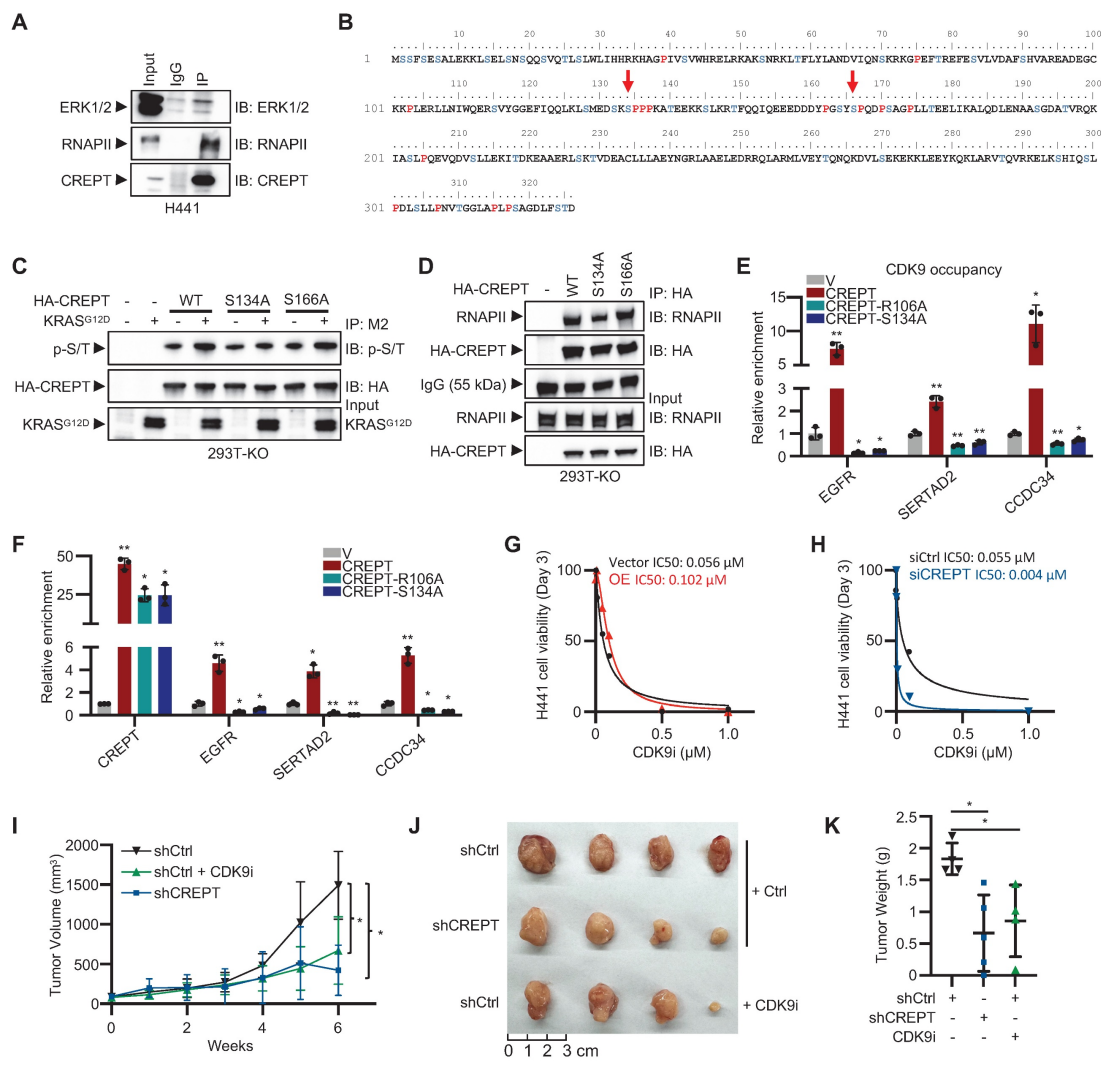
** ERK-mediated phosphorylation of CREPT at S134 facilitates CDK9 recruitment to Elk-1 target genes.** (A) Co-immunoprecipitation of CREPT and ERK in H441 cells. (B) Analysis of CREPT primary sequence identified two potential ERK phosphorylation motifs, S134-P and S166-P. (C) Western blot analysis of HA-CREPT phosphorylation in HEK-293T CREPT KO cells transfected with HA-CREPT^WT^, HA-CREPT^S134A^, HA-CREPT^S166A^, vector control, or KRAS^G12D^ using anti-HA antibody for IP and anti-phosphothreonine-proline/phosphoserine-proline (p-S/T) antibody for detection. (D) Co-immunoprecipitation assay was performed using an anti-HA antibody and samples were analyzed by Western blotting with anti-HA and RNAPII antibodies to detect HA-CREPT^WT^, HA-CREPT^S134A^, HA-CREPT^S166A^, and RNAPII. (E) ChIP-qPCR analysis of CDK9 occupancy at the promoters of *EGFR*, *SERTAD2*, and *CCDC34* in H441 shCREPT cells overexpressing CREPT^WT^, CREPT^R106A^, or CREPT^S134A^. The statistical significance of comparisons was determined relative to the vector control (V). (F) RT-qPCR analysis of *CREPT*, *EGFR*, *SERTAD2*, and *CCDC34* in H441 shCREPT cells overexpressing CREPTWT, CREPT^R106A^, or CREPT^S134A^. The statistical significance of comparisons was determined relative to the vector control (V). (G-H) IC50 values of CDK9 inhibitor (0.01-1 µM) in H441 cells with CREPT overexpression (G) or knockdown (H), compared to their respective controls. (I) Tumor volume measurements over time in xenograft models using CREPT knockdown H441 cells (shCREPT) compared with control cells (shCtrl) with or without CDK9i. (shCtrl, n = 4; shCtrl+CDK9i, n = 5; shCREPT, n = 4; shCREPT+CDK9i, n = 4) (J) Representative images of tumors collected from xenograft models at the end of the experiment. (K) Quantification of tumor weights from the xenograft models. Data are presented as mean ± SD. Statistical significance was determined by two-tailed Student's t-test. *p < 0.05, **p < 0.01.

Endogenous co-immunoprecipitation assays showed the interaction between CREPT and ERK under EGF stimulation in HEK-293T (Figure S7B). CREPT-ERK interaction was also observed in H441 cells (Figure 7A). ERK is known to preferentially phosphorylate serine/threonine residues within SP motifs (Ser/Thr-Pro). Analysis of the CREPT amino acid sequence identified two potential ERK phosphorylation sites, S134 and S166 (Figure 7B). To determine the specific phosphorylation site, we generated CREPT^S134A^ and CREPT^S166A^ mutants. Wild-type CREPT and the mutants were expressed in CREPT KO cells, with or without KRAS^G12D^ stimulation. Immunoprecipitation and subsequent immunoblotting using a phospho-SP/TP-specific antibody revealed increased phosphorylation of wild-type CREPT and S166A mutant in response to KRAS^G12D^ activation (Figure 7C; lines 3 vs 4; 7 vs 8). However, the S134A mutant showed no detectable change in phosphorylation level (Figure 7C; lines 5 vs 6), indicating that S134 is likely the ERK-mediated phosphorylation site in CREPT. Notably, CDK9 phosphorylation status remained unchanged with KRAS^G12D^ treatment, regardless of CREPT presence (Figure S7C), suggesting that ERK signaling promotes CREPT phosphorylation, not CDK9. Furthermore, immunoprecipitation assays showed that the CREPT^S134A^ mutant significantly reduced CREPT-RNAPII interaction (Figure 7D). Collectively, these results suggest that ERK phosphorylates CREPT at S134, enhancing its interaction with RNAPII.

To investigate whether disrupting CREPT-RNAPII interaction affects CDK9 recruitment to Elk-1 target genes, we generated two mutants including CREPT^R106A^ and CREPT^S134A^. While CREPT^R106A^ mutant was reported to interrupt the interaction of CREPT with RNAPII 28, we confirmed that CREPT^S134A^ mutant reduced CREPT-RNAPII interaction. ChIP-qPCR assays revealed that both CREPT^R106A^ and CREPT^S134A^ significantly reduced CDK9 occupancy at the promoters of *EGFR*, *SERTAD2*, and *CCDC34*, while wild-type CREPT markedly increased it (Figure 7E). Consistently, RT-qPCR results demonstrated that both mutants decreased, while wild-type CREPT increased, CDK9 occupancy at the promoters of *EGFR*, *SERTAD2*, and *CCDC34* (Figure 7F). These results indicate that the R106A and S134A mutations impair the ability of CREPT to enhance Elk-1 target gene expression and failed to promote CDK9 occupancy on their promoters.

Our findings suggest that CREPT and CDK9 form a complex, with CREPT undergoing phosphorylation by ERK following KRAS signaling activation. The phosphorylated CREPT subsequently facilitates the recruitment of CDK9 to RNAPII and Elk-1 occupied genes, suggesting a cooperative mechanism in transcriptional regulation. Given this intricate relationship, we hypothesized that CREPT might influence cancer cell sensitivity to CDK9 inhibitors (CDK9i), potentially impacting therapeutic efficacy. To test this hypothesis, we investigated the effects of CREPT expression levels on cellular responses to the CDK9 inhibitor BAY 1251152. Our results demonstrated a significant suppression of cell proliferation upon CDK9 inhibition. However, the degree of sensitivity to CDK9i varied depending on CREPT expression levels. CREPT overexpression conferred a modest decrease in CDK9i sensitivity, as evidenced by an elevated IC50 in OE cells (0.12 μM) compared to vector controls (0.056 μM) (Figure 7G). Reciprocally, CREPT depletion significantly increased the sensitivity of cells to CDK9i, reducing the IC50 from 0.055 µM to 0.004 µM (Figure 7H). Furthermore, CDK9i treatment markedly reduced colony formation in H441 cells, whereas CREPT overexpression partially mitigated this effect (Figure S7D).

Given that the cooperative mechanism between CREPT and CDK9, and the differential expression pattern observed in LUAD patients where CREPT is abundantly expressed in tumor cells while CDK9 expression remains relatively unchanged between tumor and adjacent non-tumor tissues (Figure S5F-S5I), we hypothesized that targeting CREPT might provide a more tumor-specific approach to inhibiting CDK9-mediated tumor growth. To validate this hypothesis, we employed a xenograft model using H441 cells to compare the inhibitory effects of CDK9i and CREPT depletion. The results showed that both CREPT depletion (shCREPT) and CDK9 inhibition (CDK9i) significantly attenuated tumor volume compared to controls (Figure 7I; Figure S7E). Consistently, CREPT depletion markedly reduced tumor size and weight relative to the control group (Figure 7J-K). Notably, the tumor-suppressive effect of CREPT depletion was comparable to that of CDK9 inhibition, suggesting that targeting CREPT could effectively recapitulate the tumor-suppressive effects of CDK9 inhibition. These findings support our hypothesis that CREPT inhibition may provide a potential alternative strategy to direct CDK9 inhibition for suppressing tumor growth, possibly with reduced systemic toxicity.

### Targeting CREPT significantly attenuates LUAD progression

**Figure 8:**
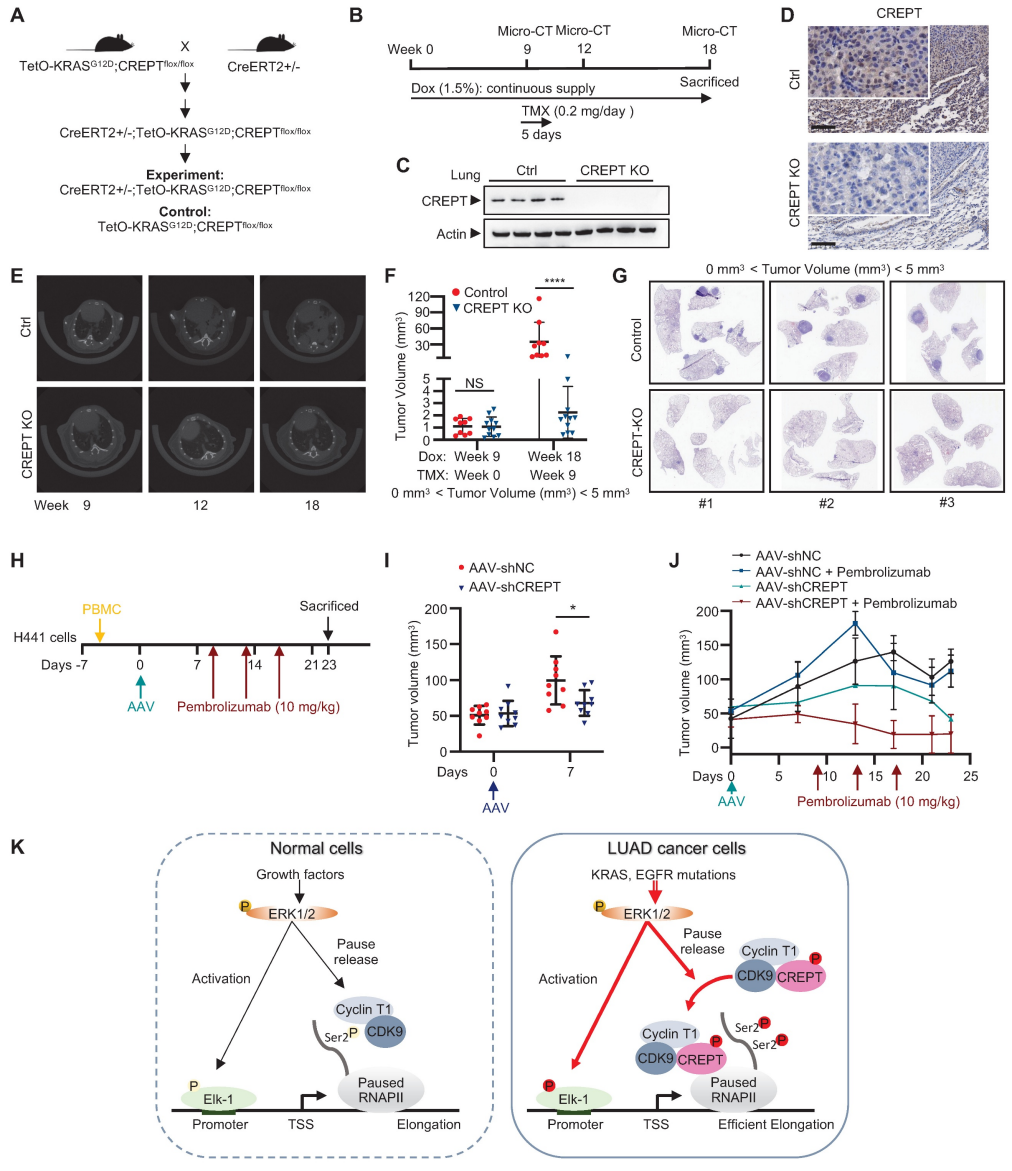
** Targeting CREPT inhibits the progression of KRAS^G12D^-driven lung adenocarcinoma *in vivo*.** (A) The establishment of CreERT2+/-;TetO-*KRAS^G12D^*;*CREPT^flox/flox^* mouse model. (B) Schematic diagram showing Dox administration to induce KRAS^G12D^ expression for tumor formation, followed by tamoxifen treatment to induce *CREPT* deletion after tumor onset, with micro-CT monitoring of tumor progression. (C) Western blot confirming CREPT KO in lung tissue from control (Ctrl) and CREPT KO mice. (D) Representative immunohistochemistry images of CREPT expression in lung sections from Ctrl and CREPT KO mice. Scale bars: 100 μm. (E) Representative micro-CT images of lungs from Ctrl and CREPT KO mice at weeks 9, 12, and 18. (F) Quantification of tumor volume from micro-CT images at weeks 9, and 18. n = 9 for Ctrl group, n = 11 for CREPT KO group. (G) H&E staining of lung sections from Ctrl and CREPT KO mice within the same rage of onset tumor volume (0 mm^3^ < Tumor volume < 5 mm^3^). (H) Schematic diagram showing humanization of NCG mice. Tumor formation was established by subcutaneous injection of 2
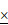
10^6^ H441 cells. AAV serotype 9 mediated CREPT shRNA (AAV-shCREPT) or control shRNA (AAV-shNC) were intravenously injected. Pembrolizumab (10 mg/kg) were administrated three times in two weeks. (I) Tumor volumes after one week after AAV administration. (J) Tumor volumes in humanized mice with more than 80% human PBMC in circulation. (K) In normal cells, CDK9/Cyclin T complex regulates RNAPII pausing and elongation in response to ERK activation. In tumor cells, CREPT acts as a scaffold protein, enhancing the interaction between CDK9 and RNAPII in response to constitutive activation of ERK. This interaction promotes CDK9 occupancy at the promoters of ERK-Elk-1 downstream target genes, facilitating Ser2 phosphorylation of paused RNAPII and efficient transcriptional elongation.

To evaluate the therapeutic potential and safety profile of systemic CREPT inhibition in LUAD treatment, we developed a genetically engineered mouse model (CreERT2+/-; TetO-*KRAS^G12D^*;*CREPT^flox/flox^*). This model enables conditional deletion of CREPT after tumor initiation, allowing us to mimic a therapeutic intervention scenario (Figure 8A). In this model, KRAS^G12D^ expression was induced by Dox administration, inducing adenocarcinoma formation. Tumor development was monitored using micro-CT. Once tumors were formed, we induced systemic *CREPT* deletion by intraperitoneal injection of tamoxifen (Figure 8B). Western blot and IHC analyses validated the efficiency of *CREPT* deletion as the CREPT protein was hardly detected after tamoxifen administration (Figure 8C-D).

Mice with varying initial tumor volumes were treated with tamoxifen to induce *CREPT* deletion. In mice with low initial tumor volumes (< 5 mm³), micro-CT analyses revealed significant suppression of KRAS^G12D^-induced adenocarcinoma growth following *CREPT* deletion (Figure 8E). A quantitative analysis showed that while tumor volumes significantly increased in control mice, they remained relatively stable in CREPT-KO mice under KRAS^G12D^ induction (Figure 8F). Notably, the majority of CREPT-deleted mice exhibited no tumor progression (Figure 8F, blue dots). Post-sacrifice examination and H&E analysis confirmed significant differences in tumor nodules between the two groups (Figure 8G, representative images from 3 of 9 mice per group). In mice with medium initial tumor volume (6-9 mm³), *CREPT* deletion resulted in a significant decrease in tumor volume after 9 weeks (Figure S8A). Consistent results were observed in mice with large initial tumor volumes (40-80 mm³). Tumor sizes in CREPT-deleted mice were substantially smaller than in control mice (Figure S8B), and H&E analysis revealed fewer tumor nodules in CREPT KO mice compared to controls (Figure S8C). Collectively, these results demonstrate that *CREPT* deletion effectively impairs the tumor progression of KRAS^G12D^ in lung adenocarcinoma *in vivo*, irrespective of initial tumor size. Furthermore, no significant differences in body weight were observed between CREPT-depleted and control groups in both female and male mice (Figure S8D-E), suggesting that systemic CREPT depletion in adult mice does not induce overt adverse effects on overall health.

We next employed a humanized mouse model to investigate whether AAV-mediated CREPT depletion could be a therapeutic strategy. H441 cells were implanted 2 days prior to PBMC introduction (Day -7), followed by administration of AAV (Day 0) and pembrolizumab on Days 9, 13, 17 (Figure 8H). Notably, tumor volumes were significantly lower in the mice injected with AAV-mediated CREPT shRNA (AAV-shCREPT) compared to those injected with control AAVs (AAV-shNC) (Figure 8I). No significant change in body weight was observed between AAV-shNC and AAV-shCREPT groups (Figure S8F). On day 9, we divided the AAV-shNC and AAV-shCREPT mice into four groups, with each group containing 4 or 5 mice that received whether a control antibody or pembrolizumab. All mice were sacrificed on day 23, and only those with more than 80% human PBMC in circulation were included in analysis. We found that mice did not response well to pembrolizumab alone (Figure 8J). However, mice treated with AAV-shCREPT showed a trend towards tumor regression after 17 days of treatment. The combination of AAV-shCREPT and pembrolizumab significantly reduced tumor volumes after a single dose of pembrolizumab. These results demonstrate that AAV-mediated CREPT silencing could be a novel therapeutic strategy, particularly when used in combination with immune checkpoint inhibitors.

## Discussion

LUAD continues to present significant therapeutic challenges, with mutations in oncogenic driver genes such as KRAS and EGFR 39. These genetic alterations result in constitutive activation of the MAPK pathway, leading to aberrant transcription of downstream genes that promote cell-cycles and proliferation 40, 41. While targeting these mutated genes with inhibitors has shown promise in clinical practice, the efficacy of current therapies remains limited due to the emergence of resistance and recurrence 42. Our study identifies CREPT, a nucleus-localized protein, as a critical participant in the transcriptional regulation of genes downstream of MAPK-ERK signaling in LUAD. We demonstrate that CREPT is highly expressed in LUAD, with its expression levels significantly correlating with patient survival and disease recurrence. Our findings provided compelling evidence that CREPT depletion substantially attenuated proliferation and tumorigenesis in LUAD cells harboring *KRAS* or *EGFR* mutations, and impaired KRAS^G12D^-induced lung adenocarcinoma formation *in vivo*. Notably, using a tamoxifen-induced *CREPT* knockout mouse model, we showed that the *CREPT* deletion could impede tumor progression even after the formation of KRAS^G12D^-induced adenocarcinoma. These findings suggest that targeting CREPT may offer a novel therapeutic strategy for LUAD.

Our study elucidates a novel mechanism by which CREPT contributes to LUAD tumorigenesis, revealing its role as a critical facilitator in the ERK-CDK9-RNAPII axis. We demonstrate that CREPT functions as a carrier protein, enhancing the interaction between CDK9 and RNAPII upon ERK activation (Figure 8K). This discovery provides new insights into the regulation of transcriptional elongation in LUAD and offers potential therapeutic targets. In normal cells, CDK9 and Cyclin T1 form the catalytic core of the P-TEFb, which is essential for efficient transcription of most RNAPII-transcribed genes under basal or stimulated conditions 43, 44, 45. Upon ERK activation, Elk-1 recruits transcriptional machinery including CDK9 and RNAPII for downstream gene transcription 13, 46, 47. CDK9 phosphorylates the CTD of paused RNAPII, promoting its release from promoter-proximal pause sites and facilitating productive elongation of transcription (Figure 8K, left panel) 45. Our study proposed an alternative mechanism of CDK9 in tumorigenesis. We found that ERK-phosphorylated CREPT promotes CDK9-RNAPII interaction, leading to increased CDK9 occupancies at ERK-Elk-1 downstream target gene promoters and elevated Ser2 phosphorylation of RNAPII. This ultimately enhances transcription and contributes to the malignant progression of LUAD (Figure 8K, right panel). Our study addresses a critical gap in understanding how CDK9 specifically facilitates Ser2 phosphorylation in tumor cells without significant elevation in expression, offering a more comprehensive model of transcriptional regulation in LUAD.

While CDK9 is crucial for tumorigenesis and CDK9 inhibition shows efficacy in the therapy of various cancers, including LUAD 15, 16, 17, we found that CDK9 is only marginally overexpressed in LUAD and its expression was not correlated with LUAD patient survival (see Figure S5E-I). This suggests that the role of CDK9 might rely on its activity rather than its expression in LUAD. Indeed, CDK9 was rarely reported to be mutated or amplified for its expression in many tumors 11. Therefore, our findings suggest that CREPT overexpression compensates for the lack of CDK9 mutations or amplifications. We validated our hypotheses by several lines of evidence including physical interaction, transcriptional co-regulation, and functional enhancement. First, we observed that CREPT expression is much strongly elevated compared with CDK9 expression (Figure S1A and Figure S5F) although their expression showed a positive correlation in LUAD (Figure S5E). Next, we observed that CREPT co-localized and directly interacted with CDK9 (Figure 5A, 5B-H, 5I, and Figure S5B-D). This physical association forms the basis for the potential of CREPT in modulating CDK9 activity. In addition, we found that CREPT promoted CDK9 recruitment to RNAPII (Figure 6G) to enhance CDK9-mediated Ser2 phosphorylation (Figure S6E). This indicates that CREPT acts as a positive regulator of CDK9 activity in modulating gene transcription. Indeed, our RNA-seq analysis revealed that CREPT and CDK9 co-regulated 409 genes (Figure 5M). Furthermore, we demonstrated that CREPT depletion reduced CDK9 occupancy on the promoters of their co-regulated genes (Figure 6H). This suggests that CREPT is able to promote CDK9 activation at the gene transcription initiation. Consistent with these biochemistry analyses, we finally provided evidence that CREPT overexpression rescued the effects of CDK9 inhibition on colony formation and CREPT depletion sensitized LUAD cells to CDK9 inhibitors (Figure 7G-H; Figure S7D). Taken together, all these results collectively suggest that CREPT overexpression boosts the CDK9 activity under the condition that CDK9 expression is not altered in LUAD. Therefore, we propose that CREPT compensates for the lack of CDK9 mutations or amplifications in LUAD.

As an amplifier of CDK9, we speculated that CREPT depletion may modulate gene transcription regulated by CDK9. However, our RNA-seq analysis indeed found that most of the DEGs after CREPT depletion had fold changes less than 2, similar to CDK9 depletion. This is quite different from previous reports including ours. This might be due to the cell model we used. Therefore, we employed GSEA, a method that examines the entire spectrum of gene expression changes rather than focusing solely on the most significantly DEGs. This approach was particularly advantageous for our dataset, where CREPT or CDK9 depletion resulted in subtle but widespread alterations across numerous genes, allowing us to capture more nuanced effects that might have been overlooked by traditional differential expression analysis. We revealed that about half of DEGs in CREPT depleted cells were also observed in CDK9 depletion, further demonstrating a cooperation between CDK9 and CREPT.

Previous studies have revealed that the R106 residue of CREPT is critical for its interaction with the CTD of RNAPII 25, 28. Our current study extends this understanding by demonstrating that the phosphorylation status of CREPT also plays a crucial role in the CREPT-RNAPII interaction. We have identified that ERK binds to CREPT and phosphorylates CREPT at the S134 residue. However, the specific binding sites between ERK and CREPT, the precise mechanism of ERK-mediated CREPT phosphorylation, and the functional consequences of these phosphorylation events in different cellular contexts remains to be explored in our future studies.

Over years, researchers have discovered numerous CDK9 inhibitors, many of which are currently in preclinical development or early-stage clinical trials 48. However, the U.S. Food and Drug Administration (FDA) has yet to approve CDK9 inhibitors, primarily due to concerns regarding their potency and specificity against tumors. The unsatisfactory clinical effects imply an incomplete comprehension of CDK9 mechanism. Our study elucidated a crucial mechanism of CDK9 regulation in LUAD progression. Additionally, we showed that elevated CREPT expression was significantly associated with poor prognosis, increased metastasis, and higher recurrence rates in NSCLC patients. These findings validated and extended the previous reports, which showed CREPT overexpression in lung cancer tissues and its negative correlation with patient outcomes 21, 22, 49. Given that CREPT exhibited strikingly higher expression in tumor tissues and its inhibition showed comparable inhibitory effects to CDK9 inhibitors on tumor growth, we hypothesize that targeting CREPT may provide an alternative and potentially more specific strategy for LUAD treatment. Moreover, we propose that this approach may have fewer on-target off-tumor effects compared to direct CDK9 inhibition in LUAD, due to the specific elevation of CREPT expression in LUAD tumor tissues. This targeted strategy could potentially offer a more selective therapeutic option with an improved safety for LUAD patients.

The clinical outcomes for CDK9 inhibitor (VIP152), demonstrated varied efficacy among patients with solid tumors 18. Therefore, we speculate that CREPT might contribute the outcomes of the therapy using CDK9 inhibitors. Our finding suggests that CREPT expression levels could serve as a potential biomarker for predicting CDK9i treatment response in LUAD patients, highlighting the need for personalized treatment strategies in this context. Given the challenges associated with the selectivity and potency of CDK9 inhibitors, targeting CREPT may be an alternative therapeutic approach. Our research demonstrates that CREPT depletion arrests cell growth across multiple LUAD cell lines, including those with EGFR mutations, KRAS mutations, EGFR inhibitor resistance, and KRAS-independence. This broad efficacy suggests CREPT as a promising target for diverse LUAD subtypes. *In vivo* studies further support this potential. Targeting CREPT showed significant anti-tumor effects in both CC10-rtTA;TetO-*KRAS^G12D^* and xenograft mouse models. More compellingly, in a humanized mouse model, AAV-mediated CREPT silencing not only halted tumor progression but also induced a trend towards tumor regression. These findings collectively underscore the robust anti-tumor activity of CREPT inhibition and its potential as a novel therapeutic strategy for LUAD.

Previous studies have shown that CDK9 inhibition sensitizes tumors to ICB therapy 12, 19. In particular, studies showed that combining CDK9 inhibition with ICB increases both T cell and antigen-presenting cell (APC) activation and function compared to ICB alone, demonstrating a function of CDK9 in modulating tumor microenvironment 19. Intriguingly, our multiplex IF analysis revealed CREPT and CDK9 expression not only in tumor cells but also in other cells within the tumor microenvironment, suggesting that CREPT may also modulate the tumor microenvironment. Given the function of CREPT as a CDK9 activator, we hypothesized that CREPT inhibition might similarly enhance ICB efficacy. Indeed, our results demonstrate a significant synergistic effect under the combination of CREPT inhibition with pembrolizumab, an antibody for ICB therapy. However, further investigation is necessary to fully elucidate the mechanisms by which CREPT inhibition modulates the tumor microenvironment and enhances ICB efficacy. Understanding these processes could lead to more effective therapies and provide new insights into overcoming resistance to immunotherapy in LUAD and other cancers. We believe these statements further strength our rational for the combination of CREPT inhibition with pembrolizumab.

We and other observed that CREPT functioned as a major regulator for variety of cancer 20. However, the mechanism through which CREPT regulates tumorigenesis in lung cancer, in particular LUAD, remains unclear. Our group and another independent research team have previously reported on CREPT expression, with the latter examining its role specifically in Calu-1 cells 21, 22. Regarding the underlying mechanisms, our studies and that of others have uncovered various regulators of CREPT across different cancer types 20. In colorectal cancer, it appeared that CREPT interacts with β-catenin, TCF4, and p300 to regulate Wnt signaling pathway 23. Additionally, our recent study demonstrated that CREPT enhanced the recruitment of p300 to phosphorylated STAT3 at the promoters of STAT3-targeted genes during tumorigenesis 50. In the present study, we have expanded our understanding of CREPT functions by elucidating its role in the regulation of RNAPII activation through its association with CDK9/Cyclin T1. Our observations suggest that CREPT serves as a critical cofactor, amplifying the effects of activated KRAS signaling in the context of LUAD development and progression.

While the established functions of CREPT in transcription termination, R-loop homeostasis, and DNA repair are well-documented, its role in LUAD progression likely encompasses additional molecular mechanisms. Our unpublished data, corroborating previous findings 27, 28, demonstrate an interaction between CREPT and Xrn2, a pivotal 5′-3′ exoribonuclease in the "torpedo model" of transcription termination. This interaction suggests that CREPT may also modulate KRAS signaling through the regulation of transcription termination dynamics. Furthermore, our ongoing investigations revealed that a significant correlation between CREPT expression and genomic instability in LUAD by analyzing the TCGA database, and CREPT depletion induced genomic instability across multiple tumor cell lines (data not shown). These observations are consistent with a previous study 26. However, elucidating the relative contributions of these diverse mechanisms to cell proliferation and LUAD progression remains challenging, as they may operate synergistically. Our study provides compelling evidence for the involvement of CREPT in the oncogenic KRAS-ERK signaling pathway. GSEA and luciferase reporter assays (Figure 3C-3H) demonstrate the regulatory role of CREPT in this pathway. Moreover, the observation that CREPT overexpression failed to promote cell growth following KRAS depletion (Figure 3I-3J) underscores its critical function in KRAS signaling. Collectively, these findings highlight the pivotal role of CREPT in modulating KRAS signaling transcription and underscore its potential as a therapeutic target in LUAD. Future studies will focus on elucidating the precise mechanisms by which CREPT influences Xrn2-mediated transcription termination and its implications in lung cancer pathogenesis. Additionally, we aim to further characterize the interplay between various functions of CREPT and their collective impact on tumor progression, potentially unveiling novel therapeutic strategies for LUAD treatment.

While our study provides evidence using LUAD cells harboring *KRAS* or* EGFR* mutations and KRAS^G12D^-driven lung adenocarcinoma, it would be valuable to investigate whether this mechanism occurs in other cancers with different oncogenic changes that consistently activate ERK. Furthermore, given that KRAS mutations are widely reported in several cancer types, exploring the role of CREPT in regulating mutation-induced activation of MAPK signaling in these other cancers remains an intriguing avenue for future research.

## Conclusion

In conclusion, our study demonstrates the elevated expression of CREPT in LUAD and elucidates its critical role in LUAD progression. We unveiled a novel mechanism wherein CREPT functions as a carrier protein for CDK9, facilitating its recruitment to paused RNAPII at the promoters of KRAS-ERK-Elk-1 downstream genes in response to ERK activation. Notably, our xenograft model revealed that depletion of CREPT yielded comparable inhibitory effects to CDK9i, underscoring the potential of CREPT as a therapeutic target. AAV-mediated CREPT silencing in humanized mice induced a trend towards tumor regression. The combination of CREPT depletion with pembrolizumab significantly enhanced tumor reduction, revealing a promising synergistic therapeutic potential. These findings collectively suggest that CREPT could serve as a promising and specific target for LUAD treatment, potentially offering an alternative therapeutic strategy for patients harboring MAPK-ERK signaling-related mutations.

## Supplementary Material

Supplementary figures and tables 1-3.

Supplementary table 4: intersection_siCREPT_819genes.

Supplementary table 5: siKRAS_downregulated_genes.

Supplementary table 6: intersection_SKsiCREPT_H441siCREPT_CDK9.

## Abbreviations

NSCLC: Non-small cell lung cancer
LUAD: Lung adenocarcinoma
LUSC: Lung squamous cell carcinoma
KRAS: Kirsten rat sarcoma proto-oncogene
EGFR: Epidermal growth factor receptor
RNAPII: RNA polymerase II
CDK9: Cyclin-dependent kinase 9
CREPT: Cell cycle-related and expression elevated protein in tumor
MAPK: Mitogen-activated protein kinase
PI3K: Phosphoinositide 3-kinase
P-TEFb: Positive transcription elongation factor
CTD: C-terminal domain
ERK: Extracellular signal-regulated kinase
OS: Overall survival
AAV: Adeno-associated virus
DEGs: Differentially expressed genes
GSEA: Gene set enrichment analysis
TCGA: The Cancer Genome Atlas
ChIP: Chromatin immunoprecipitation
IHC: Immunohistochemistry
