# Supplementary material for: CREPT promotes LUAD progression by enhancing the CDK9 and RNAPII assembly to promote ERK-driven gene transcription
Source: Theranostics. 2025 Jul 25;15(16):8337–59. doi: 10.7150/thno.115572 (PMC12374588; doi:10.7150/thno.115572)

Supporting Information

CREPT promotes LUAD progression by enhancing the CDK9 and RNAPII assembly to promote ERK-driven gene transcription

Mengdi Li[1#], Yuting Lin[1#], Jiayu Wang[1], He Yang[1], Danhui Ma[1], Ye Tian[2,3], Yi Wang[4], Liu Yang[1], Umar Farooq[5], Yinyin Wang[1], Fangli Ren[1], Jian Sheng[6], Guoqing Zhang[7], Liang Chen[8], Jun Li[9], Xiangnan Li[7*], Zhijie Chang[1,5*]

[1]State Key Laboratory of Membrane Biology, School of Basic Medical Sciences, School of Medicine, Tsinghua University, Beijing, 100084, China.

[2]Beijing Tsinghua Changgung Hospital, School of Clinical Medicine, Tsinghua University, Beijing, 102218, China.

[3]Institute for Organ Transplant and Bionic Medicine, Tsinghua University, Beijing, 102218, China

[4]Key Laboratory of Industrial Biocatalysis, Ministry of Education of China, Department of Chemical Engineering, Tsinghua University, Beijing, 100084, China.

[5]Jinfeng Laboratory, Chongqing, 401329, China.

[6]Department of science and education department, The Second Affiliated Hospital of Jiaxing University, Jiaxing, 314000, China

[7]Thoracic Surgery Department, First Affiliated Hospital of Zhengzhou University, Zhengzhou, 450052, China.

[8]Institute of Life and Health Engineering, College of Life Science and Technology, Jinan University, Guangzhou, 510632, China.

[9]Heya Pharmaceutical Technology Company, Beijing, 100176, China.

Running Title: CREPT modulates ERK-driven transcription in LUAD

# Contributed equally.

* Corresponding author:

Xiangnan Li

Tel: +86-371-66279122, Fax: +86-371-66279122, E-mail: lxn-2000@163.com

Zhijie Chang

Tel: +86 10 62785076, Fax: +86 10 62773624, E-mail: zhijiec@tsinghua.edu.cn.

Supplementary Materials 1: Supplementary Figures

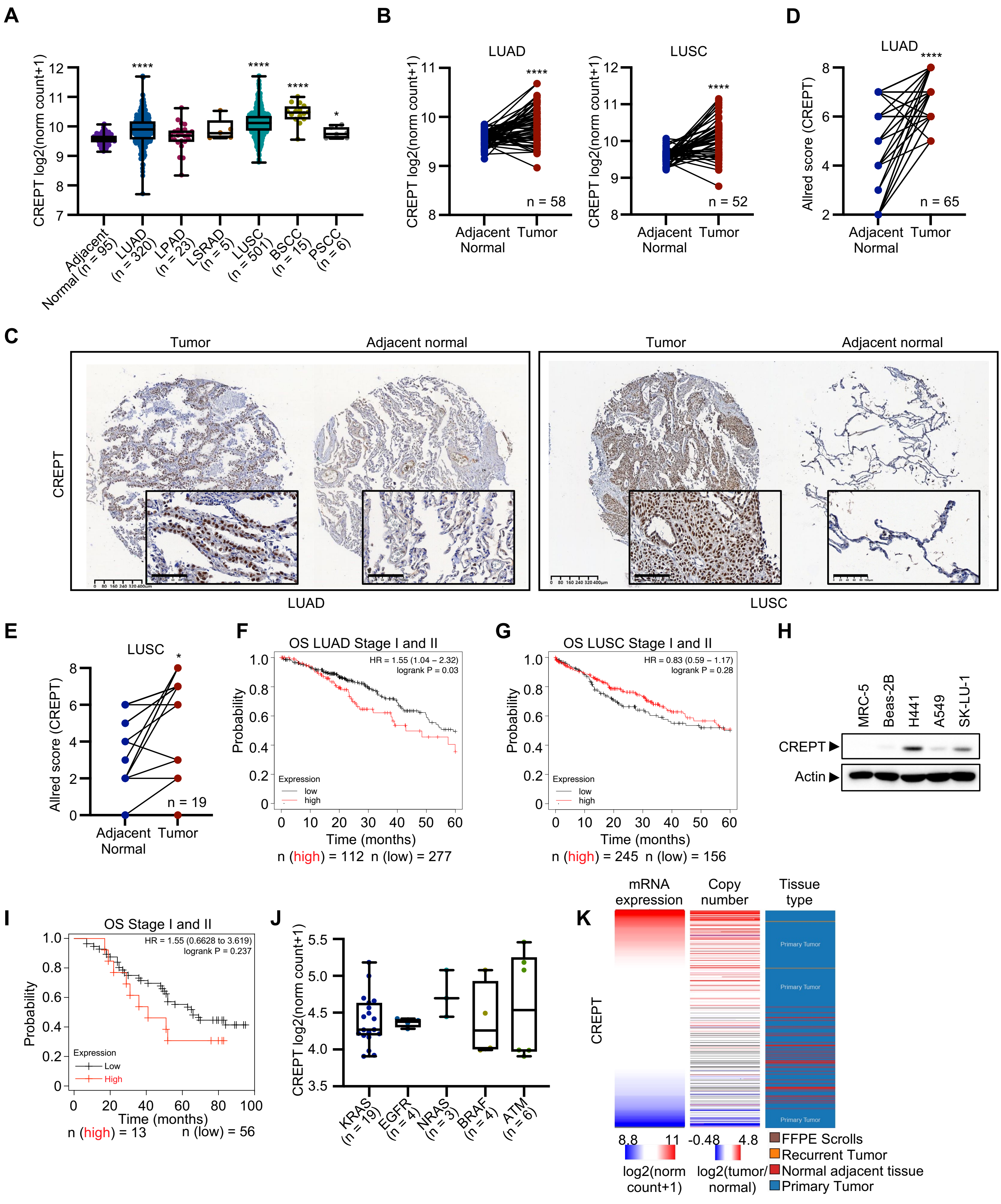

Figure S1. related to Figure 1

(A) The CREPT expression levels across different lung cancer types and adjacent normal tissues. (B) Paired analysis of CREPT expression in tumor and adjacent normal tissues of lung adenocarcinoma (LUAD) and lung squamous cell carcinoma (LUSC) patients. (C) Immunohistochemical (IHC) staining of CREPT in tumor and adjacent normal tissues from LUAD and LUSC tissue arrays. Scale bars: 400 μm (main images) and 100 μm (enlarged images). (D) Paired analysis of CREPT expression in tumor and adjacent normal tissues of LUAD patients (n = 65). (E) Paired analysis of CREPT expression in LUSC tumor and adjacent normal tissues (n = 19). (F-G) Kaplan-Meier overall survival (OS) analysis for LUAD (F) and LUSC (G) patients at stage I and II based on CREPT expression. (H) Western blot analysis of CREPT protein levels in LUAD cell lines and Beas-2B and MRC-5. (I) Kaplan-Meier overall survival analysis for stage I and II LUAD patients according to the CREPT expression level in the LUAD tissue array. (J) CREPT expression levels in LUAD cell lines with various genetic mutations, based on data obtained from the Innopedia database (innopedia.kyinno.com). (K) Analysis of TCGA data using Xena platform to visualize the correlation of CREPT mRNA levels and copy numbers in tumor and adjacent normal tissues in LUAD. Data are presented as mean ± SD. Statistical significance was determined by paired or unpaired t-test for expression comparisons. *$p < 0.05$, ****$p < 0.0001$.

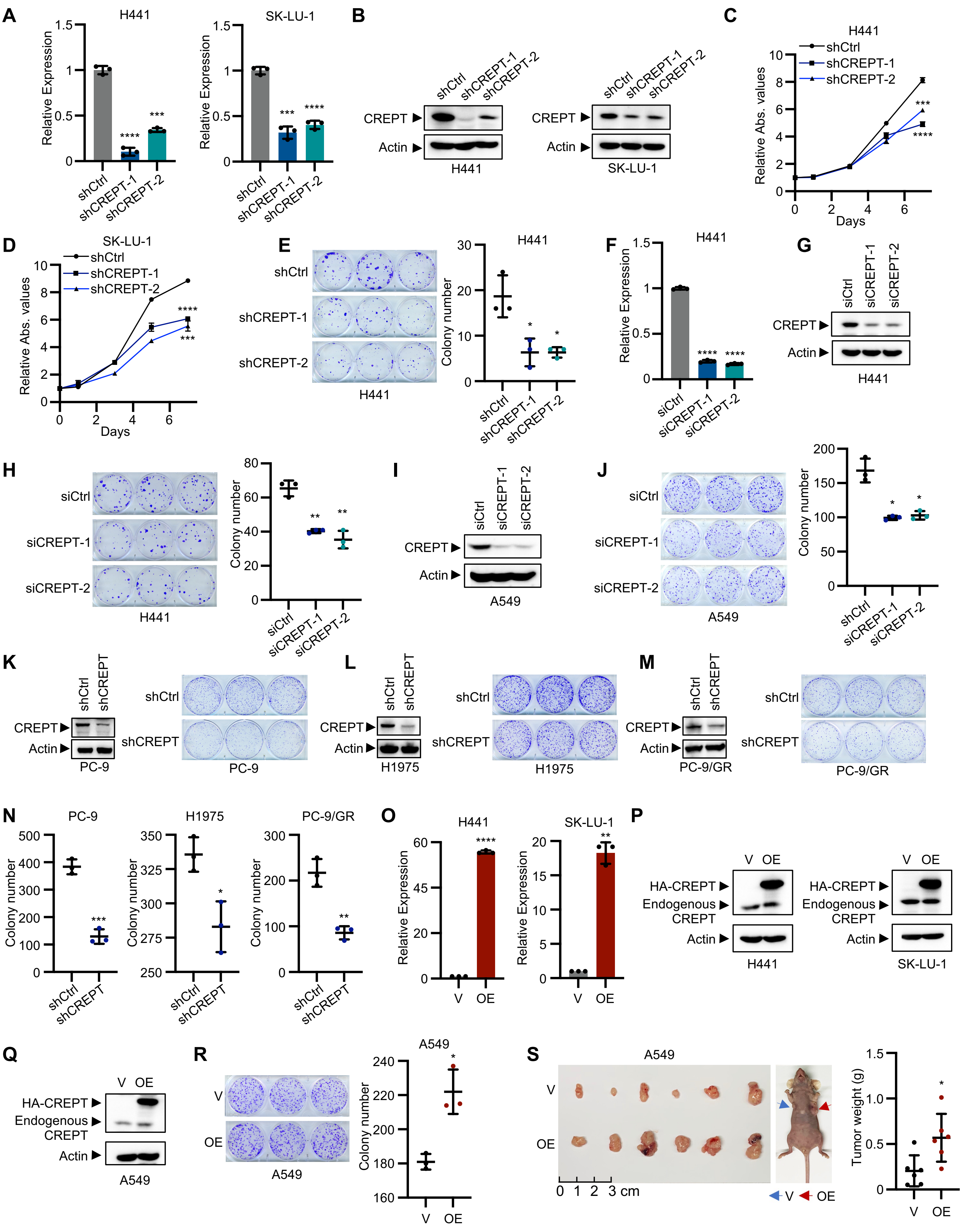

A
H441
SK-LU-1
Relative Expression
shCtrl
shCREPT-1
shCREPT-2
B
CREPT
Actin
H441
SK-LU-1
C
Relative Abs. values
Days
D
E
Colony number
F
siCtrl
siCREPT-1
siCREPT-2
G
H
I
A549
J
K
shCREPT
PC-9
L
H1975
M
PC-9/GR
N
O
V
OE
P
HA-CREPT
Endogenous CREPT
Q
R
S
0 1 2 3 cm
Tumor weight (g)

Figure S2. related to Figure 2

(A-B) CREPT mRNA (A) and protein (B) levels in H441 and SK-LU-1 cells after shRNA knockdown, assessed by RT-qPCR and Western blot, respectively. (C-D) Cell proliferation assays of H441 (C) and SK-LU-1 (D) cells with CREPT knockdown (shCREPT-1 and shCREPT-2) compared to control (shCtrl). (E) Colony formation assay of H441 cells with CREPT knockdown. Representative images (left) and quantification (right). (F-G) RT-qPCR (F) and Western blot (G) analysis of CREPT expression in H441 cells after siRNA-mediated knockdown. (H) Colony formation assay and quantitative analysis of H441 cells following CREPT knockdown using siRNAs. (I) Western blot analysis of CREPT expression in A549 cells after siRNA knockdown. (J) Colony formation assay and quantitative analysis of A549 cells following CREPT knockdown using siRNAs. (K-M) Western blot analysis of CREPT expression in PC-9 (K), H1975 (L) and PC-9/GR (M) cells after shRNA knockdown, alongside colony formation assays of these cells following CREPT depletion. (N) Quantitative analysis of colony formation numbers in K, L and M. (O-P) RT-qPCR (O) and Western blot (P) analysis of CREPT overexpression levels in H441 and SK-LU-1 cells. (Q) Western blot analysis of CREPT overexpression levels in A549 cells. (R) Colony formation assay and quantitative analysis of A549 cells with CREPT overexpression. (S) Tumor growth assay using A549 cells in nude mice and the quantitative analysis of tumor weight. Data are presented as mean ± SD. Statistical significance was determined by unpaired t-test for expression comparisons. *$p < 0.05$, **$p < 0.01$, ***$p < 0.001$, ****$p < 0.0001$.

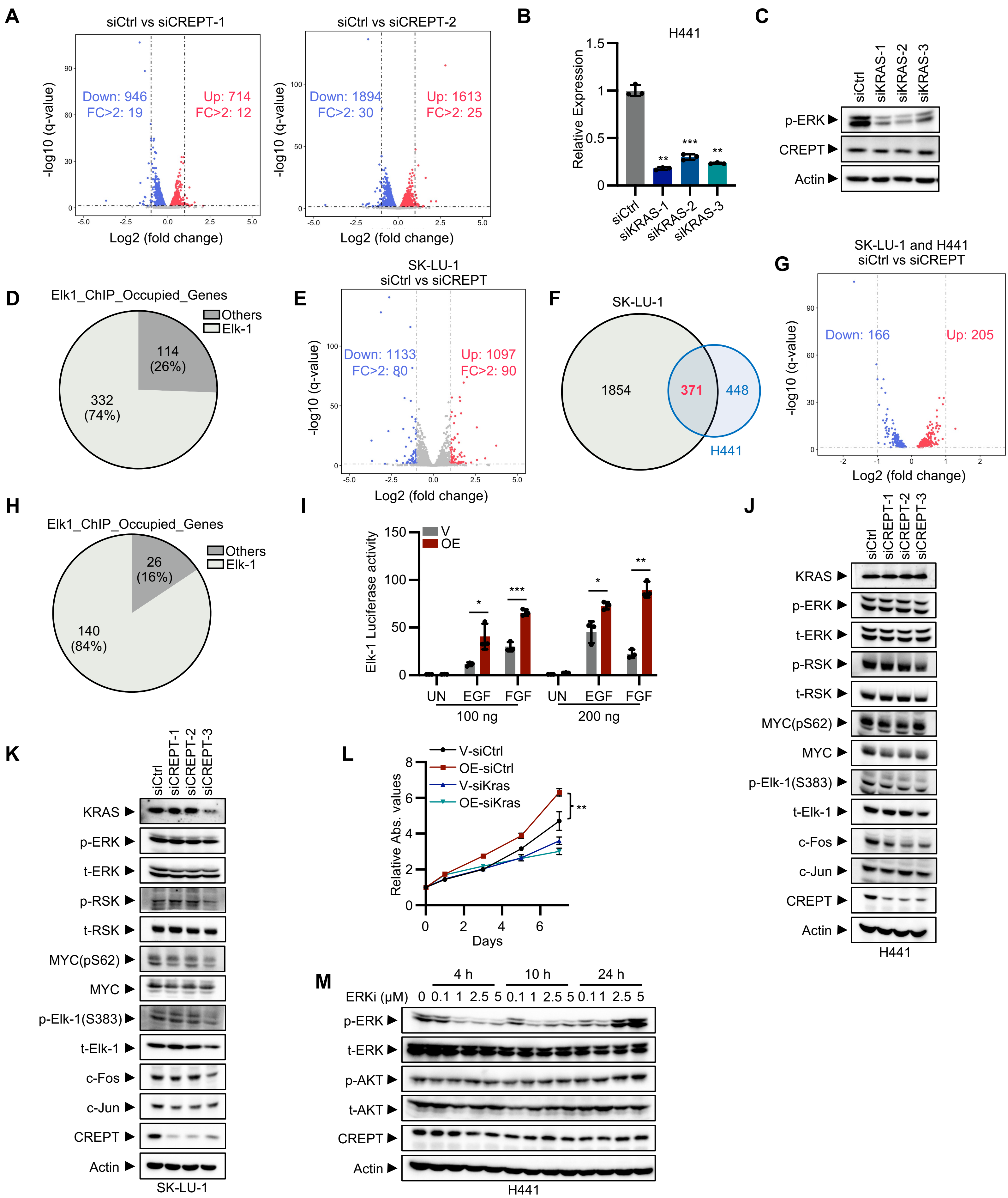
A
siCtrl vs siCREPT-1
Down: 946
FC>2: 19
Up: 714
FC>2: 12
-log10 (q-value)
Log2 (fold change)
siCtrl vs siCREPT-2
Down: 1894
FC>2: 30
Up: 1613
FC>2: 25
B
H441
Relative Expression
siCtrl
siKRAS-1
siKRAS-2
siKRAS-3
C
siCtrl
siKRAS-1
siKRAS-2
siKRAS-3
p-ERK
CREPT
Actin
D
Elk1_ChIP_Occupied_Genes
Others
Elk-1
114
(26%)
332
(74%)
E
SK-LU-1
siCtrl vs siCREPT
Down: 1133
FC>2: 80
Up: 1097
FC>2: 90
F
SK-LU-1
1854
371
448
H441
G
SK-LU-1 and H441
siCtrl vs siCREPT
Down: 166
Up: 205
H
Elk1_ChIP_Occupied_Genes
Others
Elk-1
26
(16%)
140
(84%)
I
V
OE
Elk-1 Luciferase activity
UN
EGF
FGF
100 ng
200 ng
J
siCtrl
siCREPT-1
siCREPT-2
siCREPT-3
KRAS
p-ERK
t-ERK
p-RSK
t-RSK
MYC(pS62)
MYC
p-Elk-1(S383)
t-Elk-1
c-Fos
c-Jun
CREPT
Actin
H441
K
siCtrl
siCREPT-1
siCREPT-2
siCREPT-3
KRAS
p-ERK
t-ERK
p-RSK
t-RSK
MYC(pS62)
MYC
p-Elk-1(S383)
t-Elk-1
c-Fos
c-Jun
CREPT
Actin
SK-LU-1
L
V-siCtrl
OE-siCtrl
V-siKras
OE-siKras
Relative Abs. values
Days
M
4 h
10 h
24 h
ERKi (μM) 0 0.1 1 2.5 5 0.1 1 2.5 5 0.1 1 2.5 5
p-ERK
t-ERK
p-AKT
t-AKT
CREPT
Actin
H441

Figure S3. related to Figure 3

(A) Volcano plots showing differentially expressed genes (DEGs) upon CREPT knockdown using two different siRNAs (siCREPT-1 and siCREPT-2) compared to control (siCtrl). (B) Relative mRNA expression of KRAS in H441 cells after knockdown with three different siRNAs. (C) Western blot analysis of p-ERK, CREPT, and Actin in H441 cells after KRAS knockdown. (D) The proportion of Elk1-occupied genes among 446 down-regulated genes after CREPT knockdown. (E) Volcano plot depicting DEGs upon CREPT knockdown using two mixed siRNAs in SK-LU-1 cells. (F) Venn analysis illustrating the overlap of DEGs between SK-LU-1 and H441 cells after CREPT knockdown. (G) Volcano plot showing DEGs common to both SK-LU-1 and H441 cells upon CREPT knockdown. (H) The proportion of Elk1-occupied genes among the 166 down-regulated genes affected by CREPT knockdown in both SK-LU-1 and H441 cells. (I) Elk-1 luciferase activity in response to EGF or FGF treatment at different concentrations with or without CREPT overexpression in HEK-293T cells. (J) CCK-8 assay of H441 cells with CREPT overexpression (OE) and/or KRAS knockdown (siKRAS). (K-L) Western blot analysis of factors involving in the activation of MAPK signaling pathway in H441 (J) and SK-LU-1 (K) cells after CREPT knockdown. (M) Western blot analysis of phosphorylated-ERK (p-ERK), total-ERK (t-ERK) levels, phosphorylated-AKT (p-AKT), total-AKT (t-AKT) levels, and CREPT in H441 cells in response to ERK inhibitor treatment at different concentrations (0, 0.1, 2.5, and 5 μM) over 4, 10, and 24 h. Data are presented as mean ± SD. Statistical significance was determined by unpaired t-test for expression comparisons. $^{*}p < 0.05$, $^{**}p < 0.01$, $^{***}p < 0.001$.

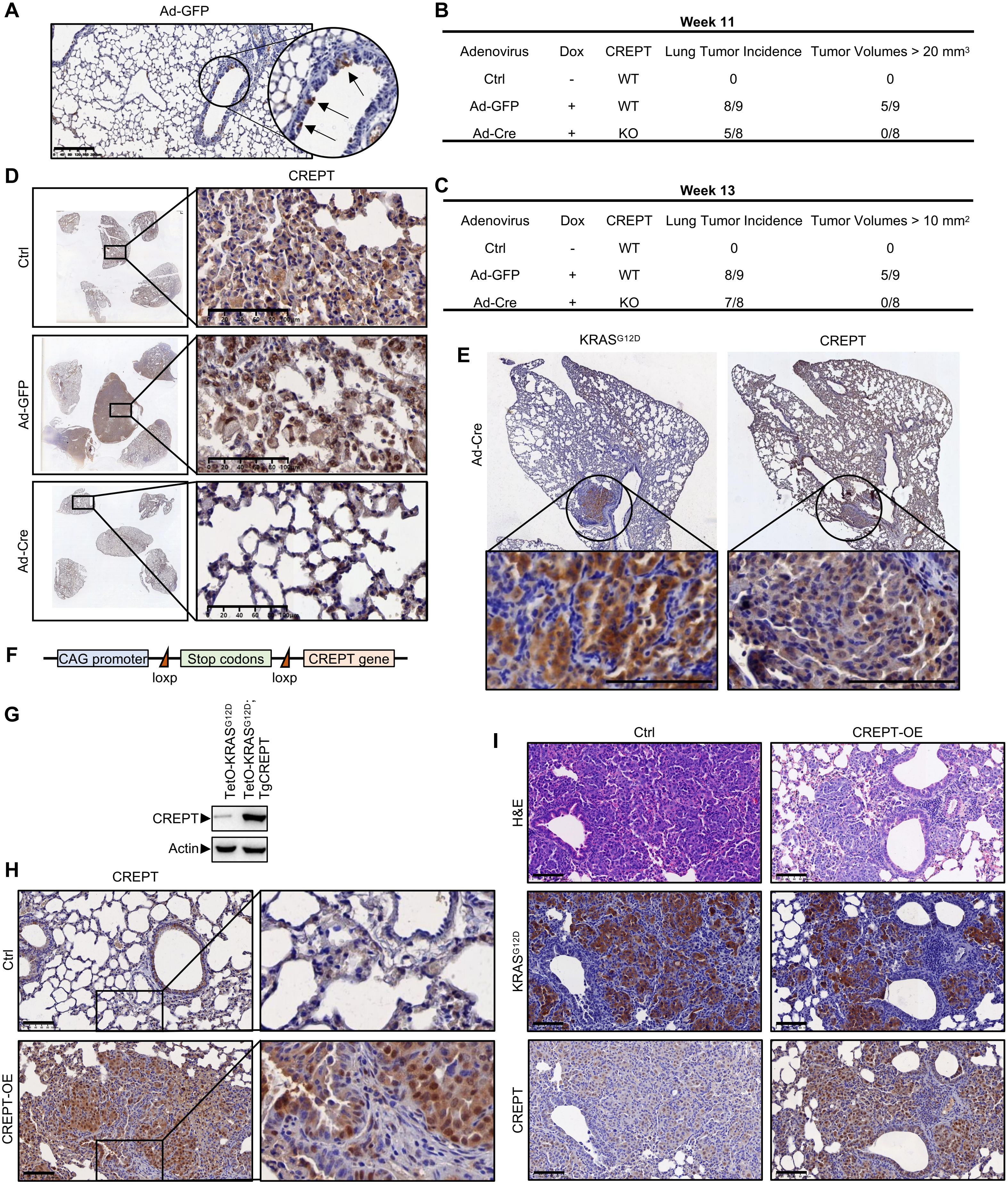

B

Week 11

Adenovirus	Dox	CREPT	Lung Tumor Incidence	Tumor Volumes > 20 mm³
Ctrl	-	WT	0	0
Ad-GFP	+	WT	8/9	5/9
Ad-Cre	+	KO	5/8	0/8

C

Week 13

Adenovirus	Dox	CREPT	Lung Tumor Incidence	Tumor Volumes > 10 mm²
Ctrl	-	WT	0	0
Ad-GFP	+	WT	8/9	5/9
Ad-Cre	+	KO	7/8	0/8

Figure S4. related to Figure 4

(A) IHC staining of GFP in Ad-CFP-induced lung tumors. Scale bar: 200 μm. (B) Table summarizing lung tumor incidence and the number of mice with tumor volumes > 20 mm^3 at week 11 in wild-type (WT) and CREPT knockout (KO) mice following doxycycline (Dox) treatment. (C) Table summarizing lung tumor incidence and the number of mice with tumor volumes > 10 mm^2 at week 13 in wild-type (WT) and CREPT KO mice following Dox treatment. (D) IHC staining of CREPT in lung sections from control mice (Ctrl) and mice treated with Ad-GFP or Ad-Cre. Enlarged images are shown on the right. Scale bars: 100 μm. (E) IHC staining of $KRAS^{G12D}$ and CREPT in the lung tumor section of mice treated with Ad-Cre. Scale bar of enlarged images: 100 μm. (F) Illustration of the loxp positions in *CREPT* gene of the TgCREPT mouse model. (G) Western blot analysis of CREPT overexpression in TgCREPT mouse. (H) IHC staining of CREPT in lung sections from control (Ctrl) and CREPT overexpression (CREPT-OE) mice. Scale bars: 100 μm. (I) IHC staining of anti-$KRAS^{G12D}$ and CREPT in lung sections from Ctrl and CREPT-OE. Scale bars: 100 μm.

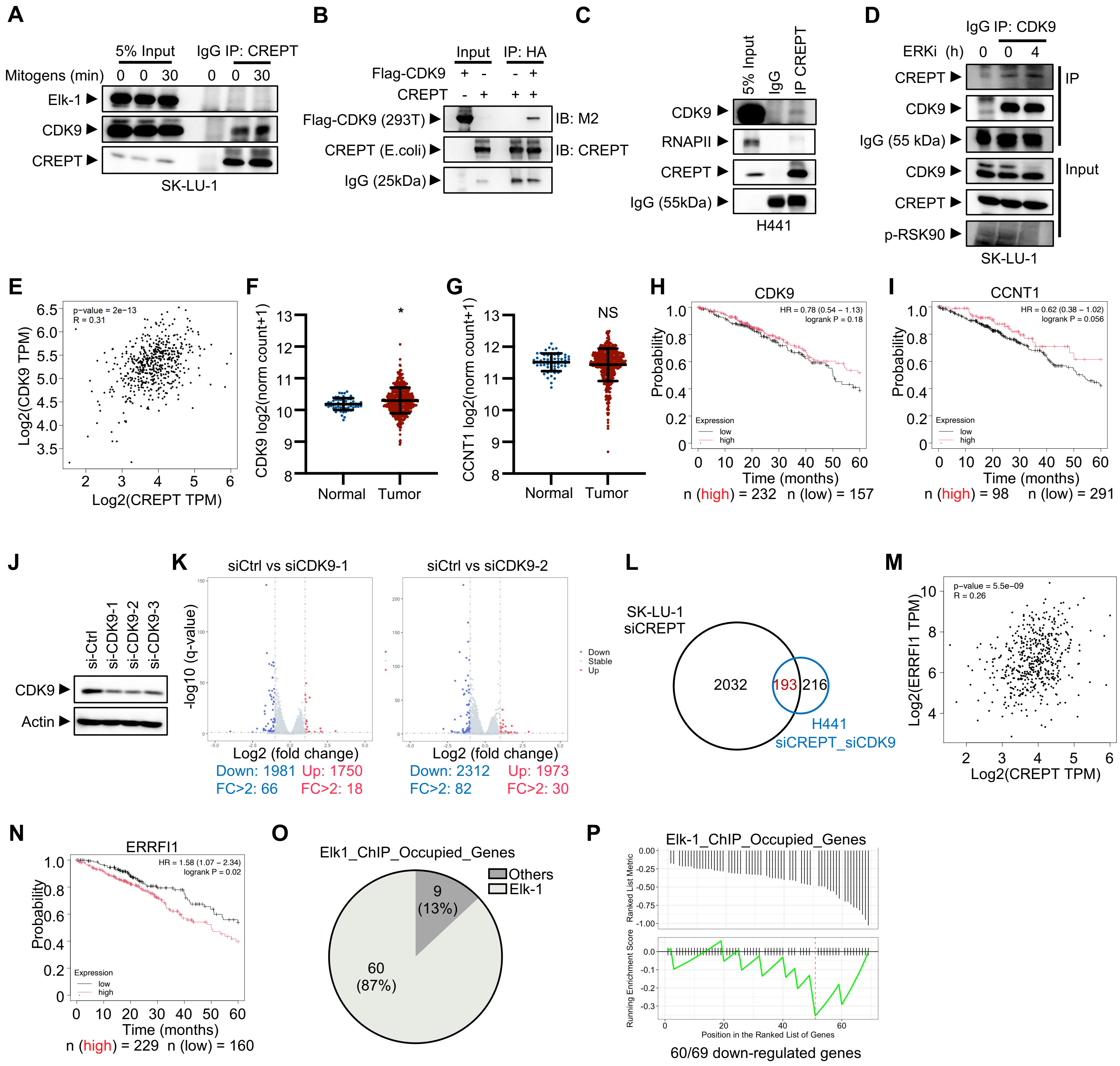

Figure S5. related to Figure 5

(A) Co-immunoprecipitation (IP) of Elk-1 and CREPT in SK-LU-1 cells under serum-starved and mitogen-stimulated conditions, with CDK9 as a positive control, analyzed by Western blot for Elk-1, CDK9, and CREPT (Input: 5%). (B) *In vitro* binding assay between purified Flag-CDK9 from HEK-293T cells and CREPT proteins from E. coli, followed by IP with CREPT antibody and Western blot analysis. (C) Endogenous co-IP of CDK9, RNAPII, and CREPT in H441 cells using CREPT antibody, analyzed by Western blot (Input: 5%). (D) Co-IP analysis of CDK9-CREPT

interaction in SK-LU-1 cells treated with ERK inhibitor for 0 or 4 h, using anti-CDK9 antibody for IP and Western blot for detection. (E) The correlation between CREPT and CDK9. (F-G) Expression levels of CDK9 (F) and CCNT1 (G) in normal and tumor tissues from the RNA-seq data of TCGA lung adenocarcinoma dataset. (H-I) Kaplan-Meier survival analysis for patients with high or low expression of CDK9 (H) and CCNT1 (I). (J) Western blot analysis of CDK9 expression after the transfection of siRNAs targeting CDK9. (K) Volcano plots showing DEGs upon CDK9 knockdown using two different siRNAs. (L) Venn analysis illustrating the overlap of 409 DEGs co-regulated by CREPT and CDK9 knockdown in H441 cells and the DEGs resulting from CREPT knockdown in SK-LU-1 cells. (M) Correlation analysis between CREPT and ERRF1 expression in LUAD samples from TCGA dataset. (N) Kaplan-Meier survival analysis for LUAD patients with high or low expression of ERRF1. (O) GSEA-style enrichment plot demonstrating enrichment of Elk-1 ChIP-occupied genes among 69 downregulated genes co-regulated by CREPT and CDK9 validated in SK-LU-1 cells. (P) The proportion of Elk1-occupied genes among the 69 down-regulated genes. Data are presented as mean ± SD. Statistical significance was determined by unpaired t-test for expression comparisons. *$p < 0.05$. NS indicates no significant difference between the groups.

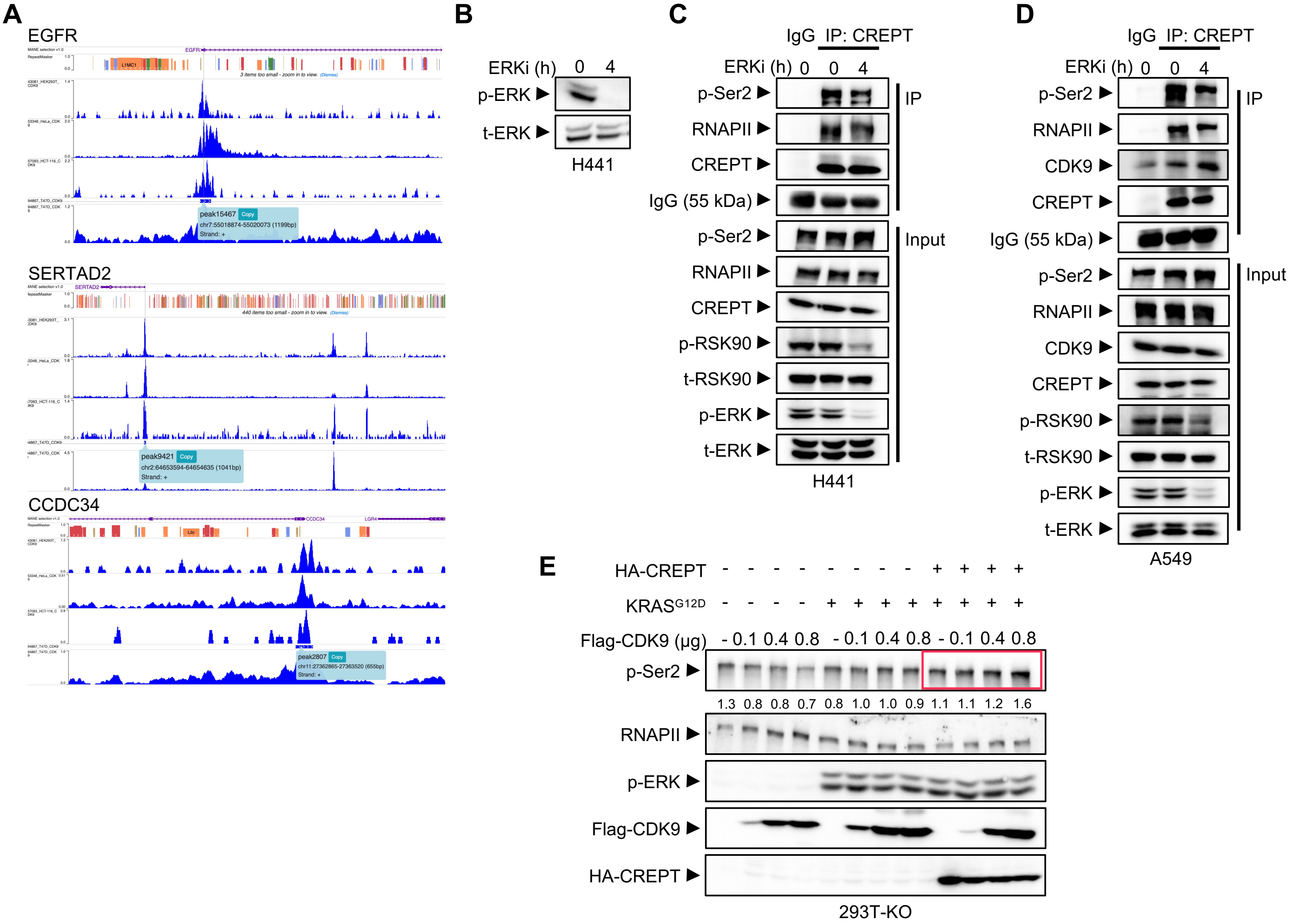

Figure S6. related to Figure 6

(A) ChIP-seq results showing the occupancy of CDK9 at the *EGFR*, *SERTAD2*, and *CCDC34* genes using ChIP-seq dataset from the Cistrome database (http://cistrome.org/db/#/). (B) Western blot analysis of ERK phosphorylation in H441 cells treated with ERK inhibitor for 0 or 4 h, validating samples used for ChIP experiments. (C-D) Co-IP and Western blot analysis of p-Ser2 and RNAPII associated with CREPT in H441 (C) and A549 (D) cells treated with ERK inhibitor for 0 or 4 h. Co-IP was performed with anti-CREPT antibody. (E) Dose-dependent effects of CDK9 overexpression on Ser2 phosphorylation in CREPT KO cells and CREPT rescued cells under KRAS^{G12D} stimulation.

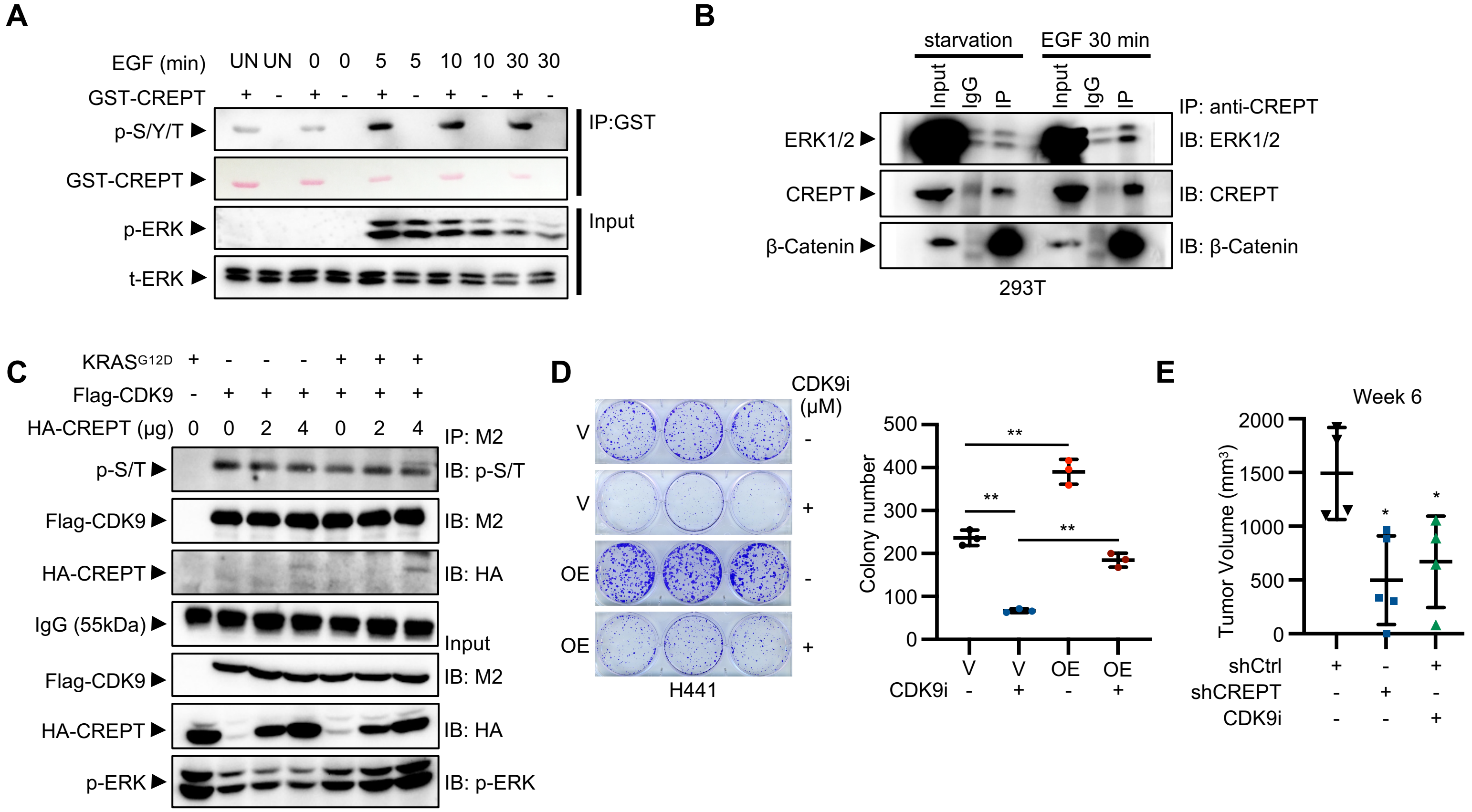

Figure S7. related to Figure 7

(A) Western blot analysis of GST-CREPT phosphorylation in HEK-293T cells under serum-starved and EGF-stimulated conditions, using an antibody anti-phosphoserine_threonine_tyrosine (p-S/Y/T) after immunoprecipitation with GST beads. (B) Co-IP and Western blot analysis of CREPT and ERK1/2 associated with CREPT in HEK-293T cells in starvation condition or EGF treatment for 30 min. (C) Western blot analysis of Flag-CDK9 phosphorylation in HEK-293T cells transfected with HA-CREPT, vector control, or KRAS^{G12D} using anti-Flag antibody (M2) for IP and anti-phosphothreonine-proline/phosphoserine-proline (p-S/T) antibody for detection. (D) Colony formation assay and quantification of CREPT-overexpressing cells versus control, with and without CDK9 inhibitor treatment. (E) Tumor volume measurements at week 6 in the xenograft model. Data are presented as mean ± SD. Unpaired t-test was used for comparisons. *p < 0.05, **p < 0.01.

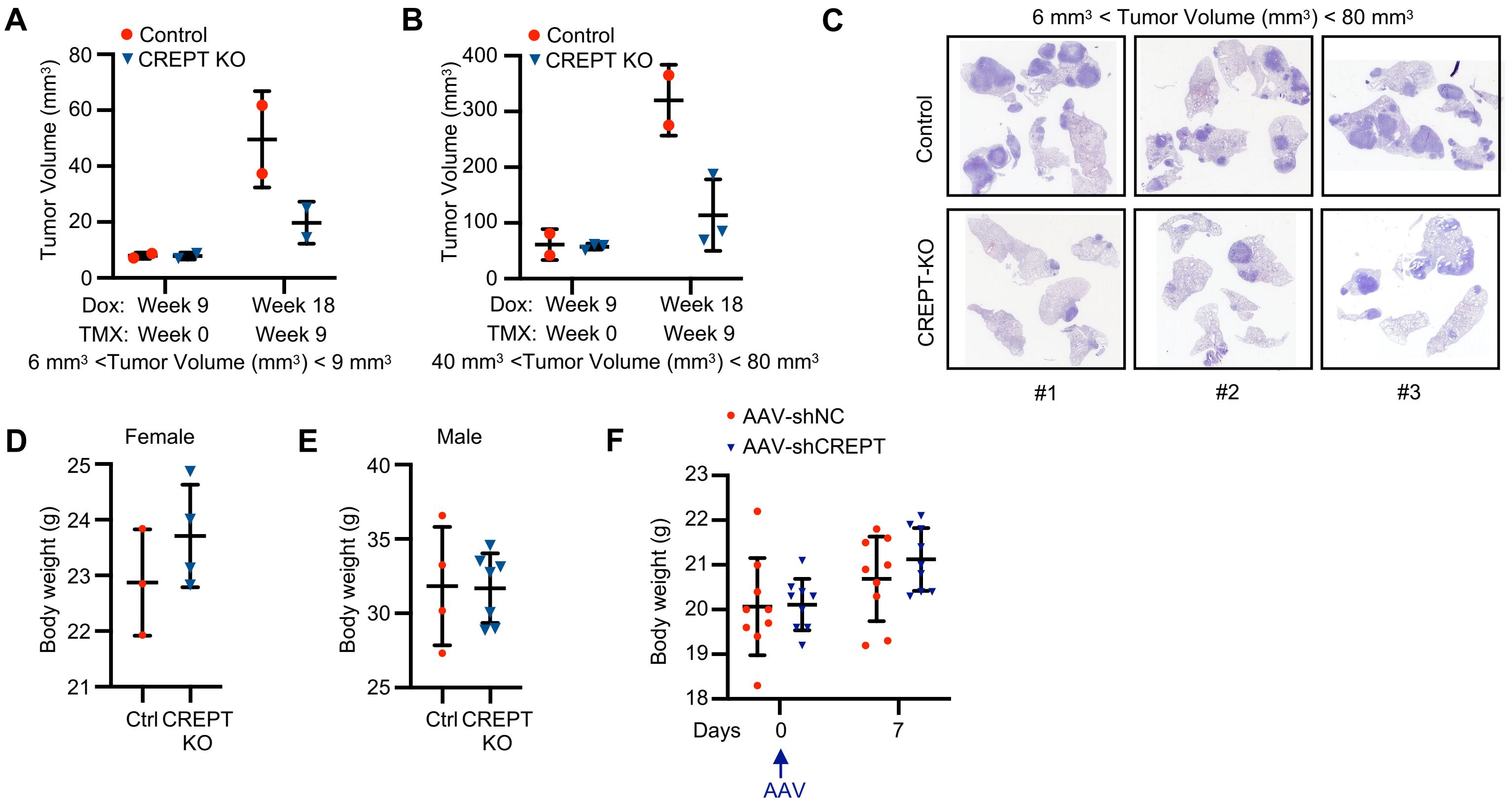

Figure S8. related to Figure 8

(A) Tumor volume measurements at weeks 9 and 18 for control and CREPT KO mice. The onset tumor volumes before CREPT KO were between 6 mm³ and 9 mm³. (B) Tumor volume measurements at weeks 9 and 18 for control and CREPT KO mice. The onset tumor volumes before *CREPT* deletion were between 40 mm³ and 80 mm³. (C) Representative histological images of tumor sections from control and *CREPT* deletion mice at week 18. The onset tumor volumes before *CREPT* deletion were between 6 mm³ and 80 mm³. (D-E) Comparison of body weights between control and CREPT KO mice at week 18, shown separately for females (D) and males (E). (F) Body weights of mice treated by AAV-shNC or AAV-shCREPT.

Supplementary Materials 2: Supplementary Tables

Table S1. The primers used in qPCR or ChIP-qPCR

**Primers**		
EGFR	F: CTTTCCTGTTTCCTTGAGATCAGC	ChIP-qPCR
	R: CCGAAGAACGAAACGTCCC	
SERTAD2	F: GGAGTGGGCGGAAACCC	
	R: GGGAGAACTTACCACATGCAG	
CCDC34	F: GACAGGCCCTCAAAGTTGT	
	R: TGCTGTTTGCAAGCTTGTTTAG	
KRAS	F: TGGTGGTGTGCCAAGACATT	RT-qPCR
	R: CACCTCACCATGCCATCTCA	
CDK9	F: TGCTGCTTAACGGCCTCTAC	
	R: ATCTCTGCCATGATGCACCC	
CREPT	F: TGTCCCTTTGGCTCATCCAC	
	R: CATCTGCCTCTCTGGCAACA	
EGFR	F: TTGCATTGATAGAAATGGGCTGC	
	R: CTGAGTGGGTCGAGGAGGTT	
SERTAD2	F: CAGAGAACGGCGGAGTCTTAG	
	R: CCTCCTTTACCCAACATATATCACA	
CCDC34	F: TCCAGTTTGACGAGGACGAC	
	R: ATTCAACCTGAGTGCTGGCG	
β-ACTIN	F: GCATCCTCACCCTGAAGTAC	
	R: AGAGGCGTACAGGGATAGCAC	

Table S2. The list of antibodies used in Western blot.

Antibody			Dilution
RNAPII p-Ser2	ab5095	Abcam	1:1000
pSer5（4H8）	ab5408	Abcam	1:1000
Elk1（E277）	ab32106	Abcam	1:1000
M2-HRP	ab49763	Abcam	1:1000
Anti-phosphoserine_threonine_tyrosine	#61-8300	Invitrogen	1:1000
Anti-phosphothreonine-proline/phosphoserine-proline antibody	ab9344	Abcam	1:1000
Rpb1-CTD（4H8）	2629	CST	1:1000
c-Fos（9F6）	2250	CST	1:500
RSK1/2/3（32D7）	9355	CST	1:1000
pRSK（Thr359/Ser363）	9344	CST	1:500
pElk-1（Ser383）	9181	CST	1:500
c-Myc（Ser62，E1J4K）	13748	CST	1:1000
c-Myc	9402	CST	1:1000
Ras	3965	CST	1:500
CDK9（C12F7）	2316	CST	1:1000
Anti-RPRD1B (anti-CREPT)	PA5-31614	Invitrogen	1:100
Erk1/2（137F5）	4695	CST	1:2000
KRAS-G12D（D8H7）	14429	CST	1:1000

HA-probe（F-7）	sc-7392	Santa	1:1000
pErk1/2（E-4）	sc-7383	Santa	1:500
β-actin（AC-15）	3554	Sigma	1:2000
Flag（M2）	F1804	Sigma	1:1000

Table S3. CREPT expression and clinicopathological features in LUAD

Variables	Numbers	Pearson r	95% confidence interval	P
Age	557	-0.008970	-0.09198 to 0.07416	0.8327
Variables	Numbers	CREPT median (log2(norm_count+1))		P
Gender				
Females	277	9.821		
Males	237	9.876, n = 237		0.1621
Smoke				
Yes	46	9.876		
No	31	9.724		0.0587
T stage				
T1	169	9.823		
T2	272	9.863		0.5263
T3	46	9.762		0.6066
T4	19	9.754		0.2170
N stage				
N0	326	9.819		
N1	95	9.863		0.8608
N2	47	9.847		0.5627
N3	2	/		/
M stage				
M0	341	9.803		
M1	25	9.978		0.0049**

Stages			
I	271	9.821	
II	120	9.864	0.6197
III	84	9.847	0.7299
IV	26	10.01, n = 26	0.0086**

Pearson's correlation coefficient (r) was utilized to evaluate the correlation between CREPT expression and age. Statistical significance was assessed using the t-test for normal distribution or Mann-Whitney U test. For TNM stages each stage was compared to T1, N0 or M0, respectively. Stages II, III, and IV were each compared against stage I. ** indicate a significance level of $p < 0.01$.

Supplementary Materials 3: Source_DATA_Extended Data

Source_DATA_Extended Data Fig. 1

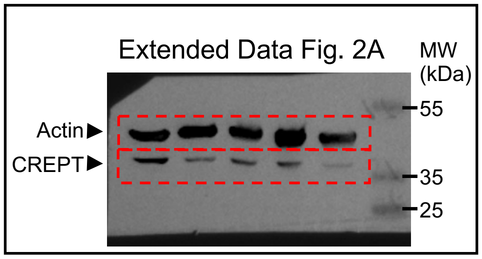

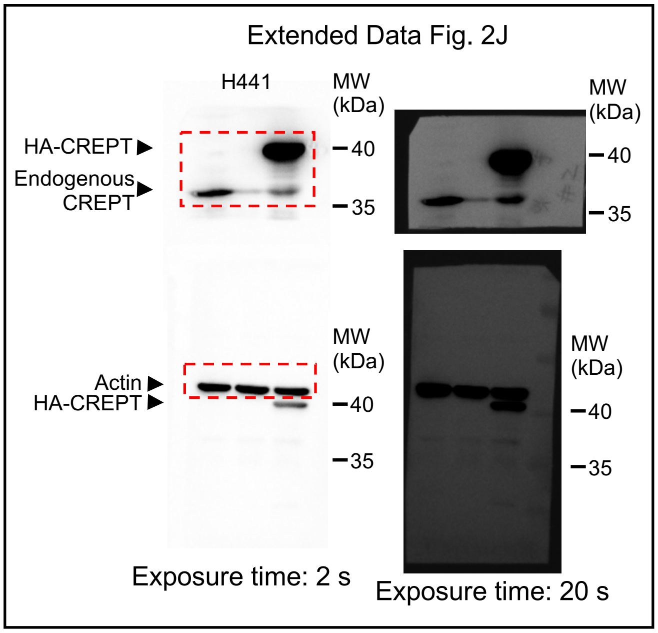

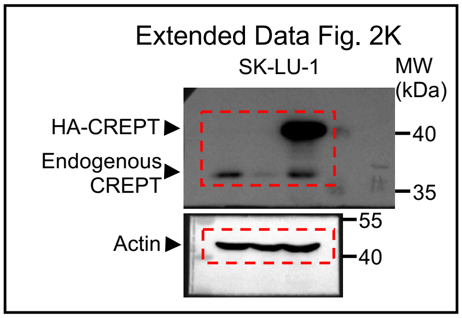

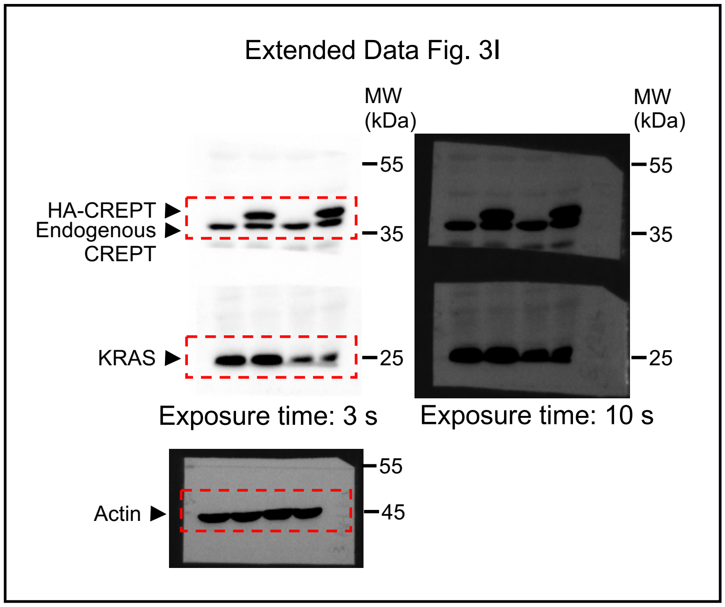

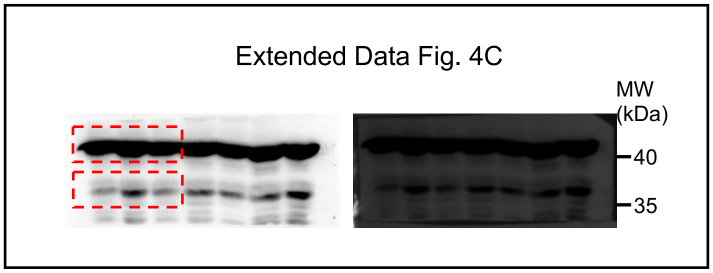

Supplement: Supplementary file 1 — Supplementary figures and tables 1-3. [file thnov15p8337s1.pdf]
